# Supramolecular Protection–Deprotection for Switching Pore‐Surface Functionality in a Crystalline Porous Organic Salt

**DOI:** 10.1002/anie.1547062

**Published:** 2026-08-05

**Authors:** Kazuki Shiga, Ryota Yamazaki, Hajime Shigemitsu, Yuuya Nagata, Norimitsu Tohnai

**Affiliations:** ^1^ Department of Applied Chemistry Graduate School of Engineering The University of Osaka Suita Osaka Japan; ^2^ Research Center for Macromolecules and Biomaterials (RCMB) National Institute for Materials Science (NIMS) Tsukuba Ibaraki Japan

**Keywords:** crystal engineering, hydrogen bonds, microporous materials, phase transitions, supramolecular chemistry

## Abstract

The precise installation of highly interactive functional groups on pore surfaces remains a central challenge in crystalline porous materials since such groups often interfere with framework formation. In this study, supramolecular protection–deprotection was performed in a porous organic salt that temporally separated framework construction from the emergence of pore‐surface functionality. During crystallization, the phenolic OH groups were protected by hydrogen bonding with dimethyl sulfoxide (DMSO), enabling the formation of a crystalline framework based on charge‐assisted hydrogen bonds. Stepwise removal of DMSO induced a two‐step single‐crystal‐to‐single‐crystal phase transition, affording first a dormant state (Form II), in which the OH groups remained protected, and subsequently an active state (Form III), in which the OH groups were exposed on the pore surface together with the emergence of porosity. The dormant state was re‐formed by exposure to DMSO or *N*,*N*‐dimethylacetamide vapor, whereas other solvents failed to induce the same structural response. In contrast, the active state showed markedly enhanced NH_3_ uptake relative to the dormant state and preferential adsorption of NH_3_ over CO_2_, N_2_, H_2_, and O_2_ under the investigated conditions. These results demonstrate that supramolecular protection–deprotection provides a useful strategy for temporally controlling pore‐surface functionality in dynamic crystalline porous materials.

## Graphical abstract

A supramolecular protection–deprotection strategy temporally separates the framework construction from pore‐surface activation in a porous organic salt. In Form I, phenolic OH groups are protected by DMSO, enabling framework formation. Stepwise single‐crystal‐to‐single‐crystal phase transformation (**SCSC**) affords a dormant intermediate (Form II) followed by an active porous state (Form III) with exposed OH groups.

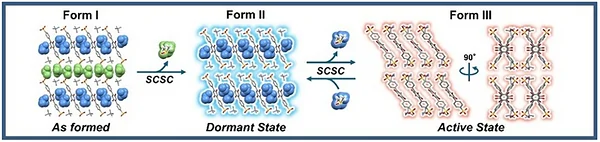

## 1 Introduction

In crystalline porous materials, the molecular recognition properties of pore surfaces are strongly governed by the type and arrangement of functional groups exposed within the pores, particularly on the pore surfaces [1, 2, 3, 4, 5, 6, 7, 8, 9, 10, 11, 12]. Therefore, the regular and accessible installation of highly interactive functional groups on pore surfaces represents a central challenge in the design of functional porous materials. Specifically, functional groups that promote guest interactions tend to participate strongly in the intermolecular interactions that govern crystallization and framework formation [3, 13, 14, 15, 16, 17]. Consequently, they can either interfere with the formation of the desired framework or become embedded into the internal bonding network instead of being exposed on the pore surface. This problem is particularly pronounced for phenolic OH groups, whose acidity and strong hydrogen‐bonding ability render them attractive for molecular recognition but also make them highly prone to intervening in structure formation itself [18], thereby hindering their selective placement on the pore surface of an ordered crystalline framework.

To address this issue, post‐synthetic modification (PSM), in which functional‐group transformation is performed after framework construction, has been extensively developed [19, 20, 21]. However, since PSM relies on covalent transformation, it can suffer from several limitations, including damage to crystallinity or porosity, heterogeneous or incomplete solid‐state reactions, and increased design complexity arising from the need to pre‐install reactive sites [19, 20, 21, 22, 23, 24]. Accordingly, a new design strategy is required that enables pore‐surface functionality to be generated and controlled after crystallization without relying on covalent transformation.

Here, we develop a supramolecular protection–deprotection strategy that addresses this challenge by temporarily rendering functional groups dormant during crystallization and releasing their function after framework formation. Specifically, in a porous organic salt (POS) bearing phenolic OH groups, the OH groups are supramolecularly protected through hydrogen bonding with dimethyl sulfoxide (DMSO) during crystallization. This decoupled the OH groups from framework formation while allowing construction of a crystalline framework through charge‐assisted hydrogen bonds (CAHBs) between ammonium ions and sulfonate groups [13, 14, 15, 16, 25, 26, 27, 28]. Subsequent heating induced a two‐step single‐crystal‐to‐single‐crystal (SCSC) phase transition [9, 29, 30, 31, 32, 33, 34] and afforded a dormant state (Form II), in which the OH groups are protected, followed by an active state (Form III), in which the OH groups are exposed on the pore surface. This strategy is designed to achieve temporal separation between framework construction and the emergence of pore‐surface functionality by using reversible hydrogen‐bond‐based supramolecular interactions rather than covalent protecting‐group chemistry. Reformation of the dormant state by exposure to specific solvent vapors demonstrated both one‐way activation and reversible control over the functional state of the pore surface [35]. Furthermore, ammonia (NH_3_) adsorption measurements revealed that the active state, in which the OH groups were exposed on the pore surface, exhibited a pronounced preference for NH_3_, an important target for selective capture and recovery [36, 37, 38, 39, 40, 41, 42, 43]. Comparison with the dormant state further demonstrated that this NH_3_‐responsive adsorption function can be switched through supramolecular protection–deprotection.

## 2 Results and Discussion

### 2.1 Structural Design and Stepwise SCSC Transformation

#### 2.1.1 Supramolecular Protection of the OH Groups by DMSO

Based on a supramolecular functional group protection strategy, a porous organic salt was designed from 2′,5′‐dihydroxy‐[1,1′:4′,1″‐terphenyl]‐4,4″‐disulfonic acid (HQDPS), a phenolic disulfonic acid bearing two OH groups, and *tert*‐butylamine (*t*BuA) (Figures S1–S5). Recrystallization of the resulting crude HQDPS/*t*BuA salts from DMSO afforded Form I, in which the OH groups were preferentially hydrogen‐bonded to DMSO molecules and therefore did not participate in framework construction (Figure 1, Figures S6–S7, Table S1) [44]. Instead, the framework itself was assembled through charge‐assisted hydrogen bonds (CAHBs), in which the ammonium cation (*t*BuAH*^+^*) acted as the proton donor and the sulfonate group as the proton acceptor. Specifically, the phenolic OH groups were preferentially captured by DMSO and were thus effectively excluded from CAHB network formation, resulting in the selective formation of Form I. Therefore, DMSO serves as both a recrystallization solvent and a protecting molecule that temporarily isolates the OH groups from framework formation [45].

**FIGURE 1:**
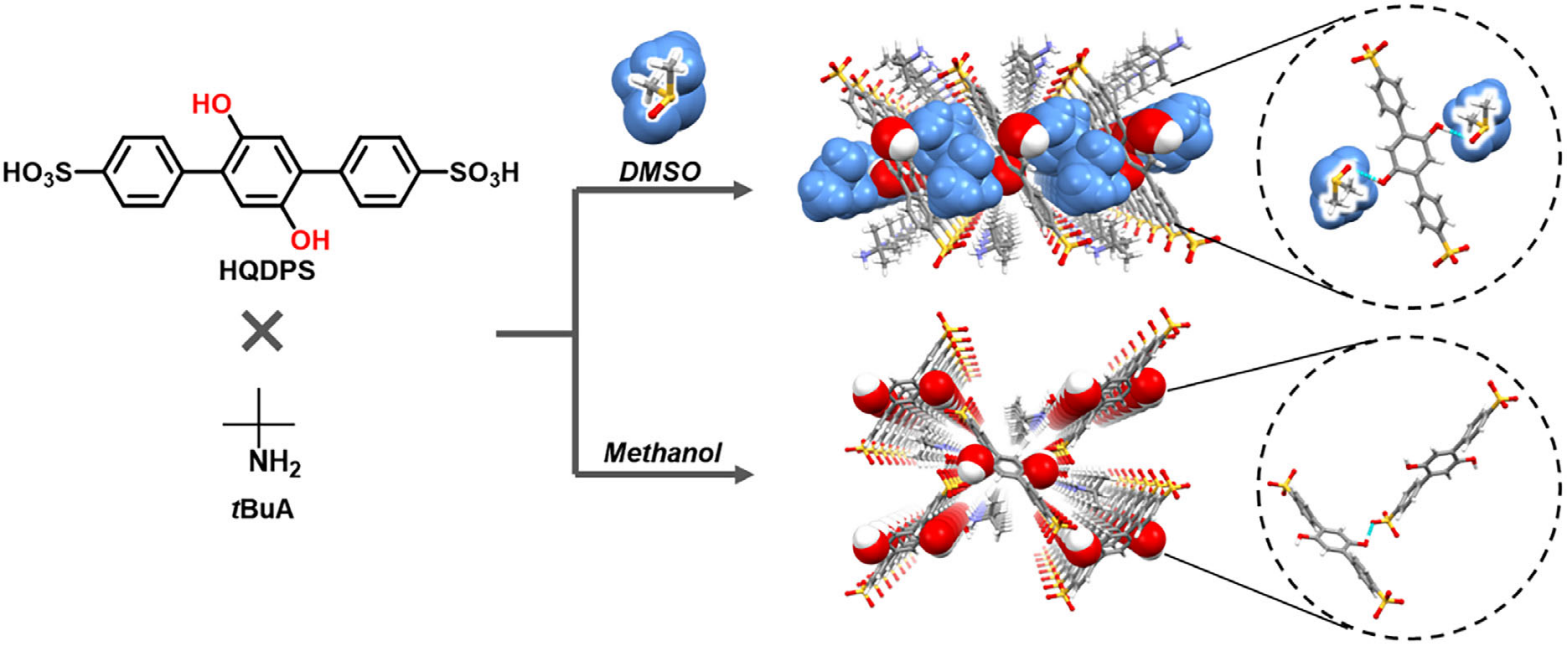
Schematic illustration of the present design strategy and comparison with the structure obtained from methanol recrystallization.

This interpretation was supported by comparison with a control experiment performed using methanol. During recrystallization from methanol, the OH groups directly formed hydrogen bonds with the sulfonate groups, yielding a crystal structure in which the OH groups were incorporated into the internal framework (Figure 1 and Figure S8). Thus, under conditions in which the OH groups remained unprotected by DMSO, they again participated in framework formation. Accordingly, Form I should not be viewed simply as a crystal phase obtained in the presence of DMSO, but rather as the outcome of supramolecular protection by DMSO, which separates the CAHB network responsible for framework construction from the OH groups that serve as functional sites. These results indicate that temporary protection of a specific functional group can separate its role in framework formation from its role as a functional site.

Subsequently, heat treatment of Form I induced a two‐step SCSC phase transition accompanied by the stepwise removal of DMSO and progressive deprotection of the OH groups. Thus, after crystallization in a state where the OH groups were temporarily decoupled from framework formation, the system passed through a dormant state (Form II) while retaining crystallinity, ultimately affording an active state (Form III) in which the OH groups were exposed on the pore surface. This behavior clearly confirms that the present strategy achieves temporal separation between the role of the OH groups in framework construction and their role as functional pore‐surface sites.

To further support the interpretation that DMSO acts as a supramolecular protecting molecule for the phenolic OH groups, we also examined a methoxy‐substituted analogue, TPDS‐OMe_2_/*t*BuA, in which the phenolic OH groups are covalently masked as methoxy groups. Recrystallization of TPDS‐OMe_2_/tBuA under conditions similar to those used for HQDPS/*t*BuA afforded two crystalline polymorphs that were structurally related to HQDPS/tBuA Form I (Figure S9). In both structures, DMSO molecules were hydrogen‐bonded to the *t*BuAH*^+^* cations, and the sulfonate–ammonium charge‐assisted hydrogen bonds formed a one‐dimensional ladder‐type network. Thus, the DMSO‐protected state of HQDPS/*t*BuA is structurally analogous to a covalently O‐methyl‐masked state, supporting the assignment of DMSO as a supramolecular protecting molecule for the phenolic OH groups.

#### 2.1.2 First SCSC Step: Selective Removal of DMSO Hydrogen‐Bonded to the *t*BuAH*^+^* Cations

Upon heating Form I at 100°C for 1 h, the DMSO molecules hydrogen‐bonded to the ammonium cations (*t*BuAH*^+^*) were selectively removed, while those hydrogen‐bonded to the OH groups were retained, resulting in conversion to Form II (Figure 2, Table S1). Structural analysis confirmed that the multiple DMSO molecules present in Form I were not equivalent; rather, they exhibited distinct behavior depending on their local binding environments. Concomitantly, the CAHB network was reorganized from a one‐dimensional (1D) ladder structure into a two‐dimensional (2D) sheet structure (Figure 2). Additionally, removal of the DMSO molecules hydrogen‐bonded to *t*BuAH*^+^*, which occupied the space between the 1D hydrogen‐bonded chains, promoted rearrangement of the sulfonate–ammonium interactions, leading to expansion of the network from a 1D structure in Form I to a 2D structure in Form II. In contrast, the OH groups remained protected by DMSO. Therefore, the first SCSC transformation should be regarded not as deprotection of the OH groups, but as the formation of the dormant state (Form II), in which the OH groups remain protected while structural reorganization proceeds.

**FIGURE 2:**
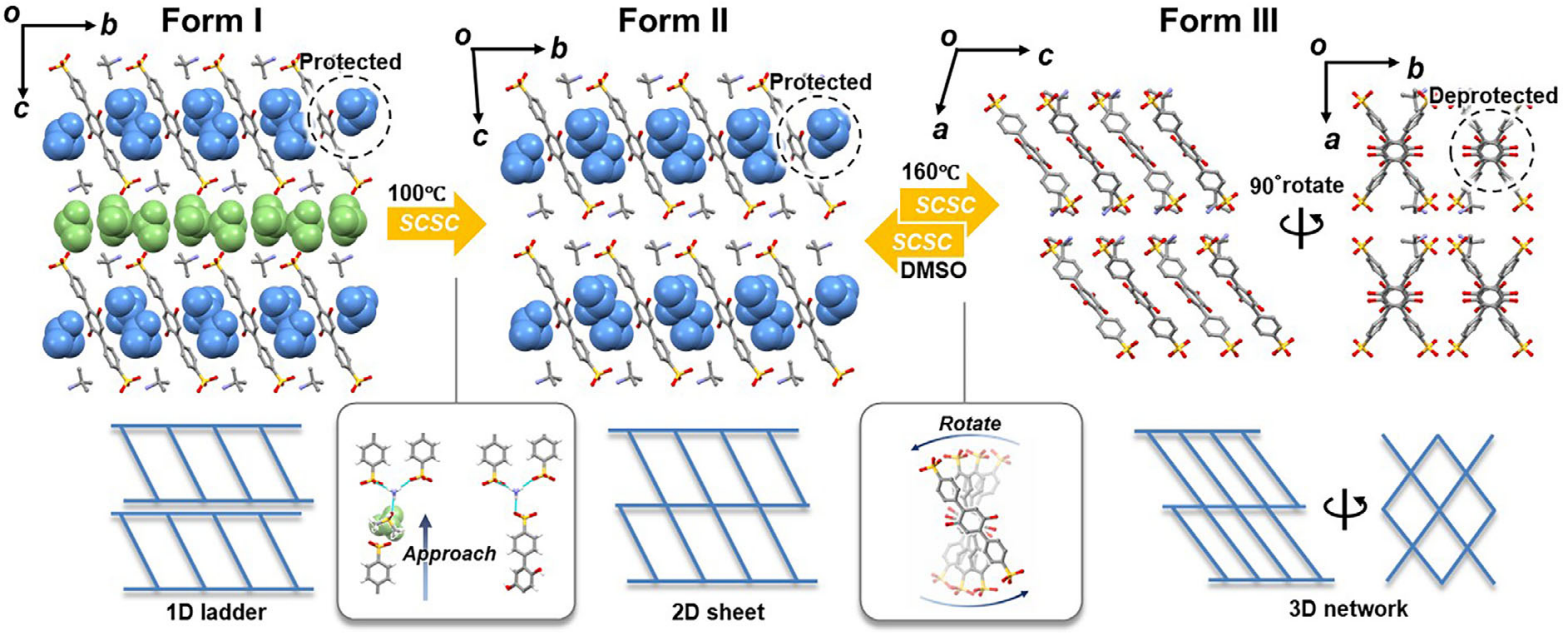
Two‐step SCSC phase transition induced by the stepwise removal of DMSO. Structural transformation from Form I to the dormant state Form II and then to the active state Form III, together with the accompanying reorganization of the CAHB network.

This interpretation was also supported at the bulk level by powder x‐ray diffraction (PXRD) and thermogravimetric analysis (TGA) (Figure 3). Specifically, Form II exhibited a diffraction pattern clearly distinct from that of Form I and in good agreement with the simulated pattern, demonstrating that the first heating step generated a single new phase (Figure 3a). In TGA, the low‐temperature weight‐loss component observed for Form I disappeared in Form II, consistent with the partial removal of DMSO during the first heating step (Figure 3b). Taken together, these results confirm that, in the first SCSC step, DMSO molecules occupying the interlayer space of the 1D ladder‐type CAHB network were selectively removed while the OH groups remained protected to afford the dormant state Form II.

**FIGURE 3:**
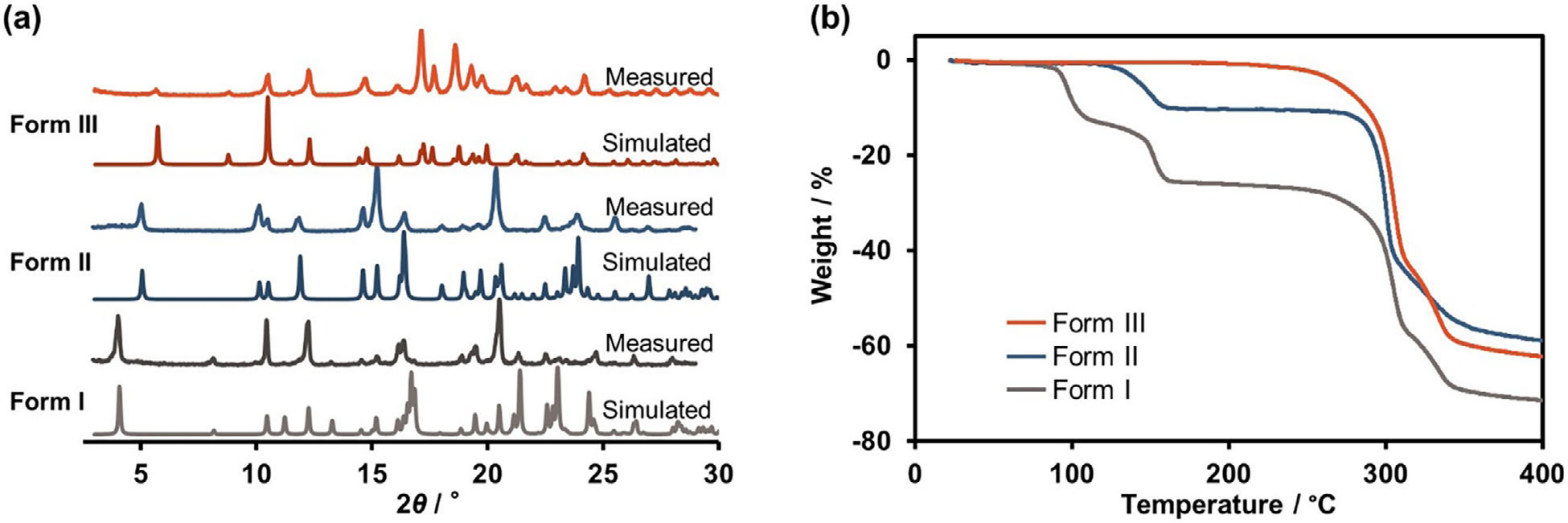
Bulk characterization of each activation stage. (a) Experimental and simulated PXRD patterns of Forms I–III. (b) TGA curves of Forms I–III.

#### 2.1.3 Second SCSC Step: Removal of the OH‐Protecting DMSO Molecules

Upon further heating Form II at 160°C for 1 h, the DMSO molecules protecting the OH groups were removed, yielding Form III (Table S1), wherein the OH groups were exposed on the pore surface (Figure 2). In this second structural transformation, the OH–DMSO interactions retained in Form II were disrupted, and the previously protected OH groups became exposed for the first time. This step therefore corresponds not merely to residual solvent removal but also to a structural transition accompanied by the emergence of functional sites.

Notably, this deprotection step was accompanied by a more extensive framework reorganization. As shown in Figure 2, removal of the DMSO molecules protecting the OH groups caused substantial rotation and displacement of the sulfonate groups, and the CAHB network changed from the 2D sheet structure of Form II to the three‐dimensional (3D) network of Form III. Consequently, Form III exhibited 1D channels with an apparent aperture of approximately 6.7 × 4.0 Å, as estimated from the final crystallographic dataset. The solvent‐accessible void volume calculated using PLATON was 294.8 Å^3^ per unit cell, corresponding to a void fraction of 17.3%. The porosity of Form III should therefore be understood not as arising from simple activation of pre‐existing voids, but as being generated through the second SCSC step, which completes OH deprotection and reorganizes the entire crystal. Details of the pore‐size evaluation and gas‐sorption‐derived analysis are provided in the Supporting Information.

This interpretation was again supported by the bulk PXRD and TGA results (Figure 3). Specifically, the diffraction pattern obtained for Form III clearly differed from that of Form II and was in good agreement with the simulated pattern, indicating that the second heating step generated another new crystal structure (Figure 3a). In TGA, the weight‐loss component observed for Form II disappeared in Form III, consistent with the removal of the remaining DMSO during the second heating step (Figure 3b). Notably, despite the large molecular displacements, high crystallinity was maintained in Form III. This observation suggests that the structural transformation proceeds not through local collapse but via a cooperative reorganization extending throughout the crystal [46, 47].

### 2.2 Reverse Phase Transition Induced by Vapor Exposure and Supramolecular Reprotection

#### 2.2.1 Reprotection of the OH Groups by DMSO Vapor

Upon exposure to DMSO vapor, Form III underwent a reverse phase transition to Form II, even at ∼25°C [48]. Specifically, when bulk crystals of Form III were exposed to DMSO vapor at ∼25°C for 24 h, the PXRD pattern became consistent with the simulated pattern for Form II, indicating structural conversion from Form III to Form II (Figure 4a). This result shows that the active state obtained by thermal activation can respond to a mild stimulus such as solvent vapor while retaining crystallinity.

**FIGURE 4:**
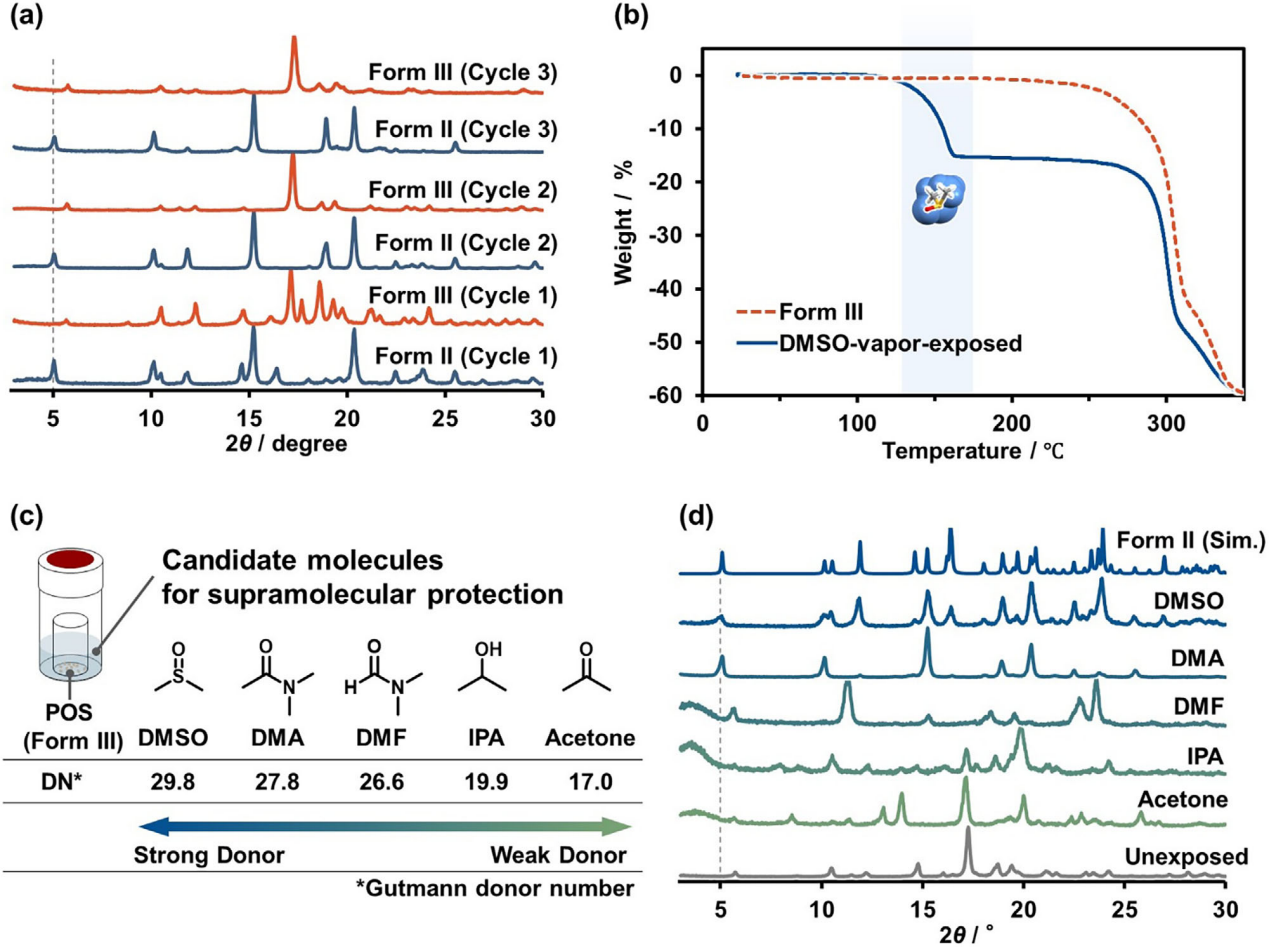
Vapor‐induced reprotection and molecular selectivity of the reverse phase transition. (a) PXRD patterns showing DMSO‐vapor‐induced reprotection (Form III → Form II) and thermal deprotection (Form II → Form III) over three cycles. (b) TGA curves of Form III before and after exposure to DMSO vapor. (c) Candidate molecules for supramolecular reprotection together with their Gutmann donor numbers. (d) PXRD patterns of Form III after exposure to various solvent vapors.

This transformation was also supported by TGA. After exposure to DMSO vapor, a new weight‐loss feature appeared at ∼150°C, which was absent in Form III before vapor exposure (Figure 4b). This behavior was consistent with the TG profile of Form II shown in Figure 3b, suggesting that DMSO vapor exposure did not merely lead to surface adsorption of the solvent but rather re‐formed the dormant state containing DMSO. Taken together, the PXRD and TGA results indicate that this vapor response should be understood not as simple DMSO uptake by Form III, but as a structural transformation to Form II accompanied by reprotection of the OH groups.

Notably, this vapor‐induced reprotection (Form III → Form II) and thermally induced deprotection (Form II → Form III) were repeatedly observed over at least three cycles (Figure 4a). After each cycle, the PXRD patterns retained the characteristic peaks of Forms II and III, respectively, without any marked loss of crystallinity. These results demonstrate that the dormant and active states can be reversibly interconverted in response to DMSO vapor and heat [35]. Thus, the present system enables reversible switching between dormant and active pore‐surface states through repeated protection–deprotection.

#### 2.2.2 Selectivity of the Reprotecting Molecules

The phase transition from Form III to Form II showed clear selectivity toward specific vapor molecules. Upon exposure of Form III to acetone, 2‐propanol, *N*,*N*‐dimethylformamide (DMF), and *N*,*N*‐dimethylacetamide (DMA) vapor under conditions analogous to those used for DMSO (Figure 4c), only DMA induced structural conversion to Form II, whereas no pronounced phase transitions were observed for the other solvents (Figure 4d). This result demonstrates that Form III exhibits structural response only to limited guest molecules.

The selectivity is likely related to the electron‐pair‐donating ability of the reprotecting molecules, namely, their hydrogen‐bond acceptor ability toward the phenolic OH groups. Indeed, DMSO and DMA, both of which induced reprotection, possess relatively high donor numbers (Figure 4c) and can act as strong hydrogen‐bond acceptors for phenolic OH groups [49, 50]. In contrast, no structural transformation was observed for molecules with lower donor numbers, such as 2‐propanol and acetone. This trend suggests that a certain level of electron‐pair‐donating ability is involved in triggering reprotection.

This interpretation was further supported by recrystallization of the HQDPS/*t*BuA salt from DMA. The resulting crystal structure was closely related to Form II, and the phenolic OH groups were supramolecularly protected through hydrogen bonding with DMA (Figure S12). Thus, DMA can also act as a supramolecular protecting molecule for the phenolic OH groups, consistent with the DMA‐induced reprotection observed in the vapor‐exposure experiment. This comparison also indicates that DMA mainly stabilizes a Form II‐like reprotected state rather than reproducing all the local interactions characteristic of the DMSO‐containing Form I.

Interestingly, DMF did not induce structural conversion to Form II despite having a donor number close to that of DMA (Figure 4d). Therefore, although an effective boundary may exist between DMA and DMF in terms of the ability to induce reprotection, the present response cannot be explained solely by donor number. In other words, for a reprotecting molecule to induce conversion to Form II, both sufficient hydrogen‐bond‐accepting ability and local compatibility with the interaction sites remaining on the pore surface after deprotection appear necessary.

The observed selectivity toward reprotecting molecules is important from the perspective of both material function and design strategy. The fact that Form III distinguishes between selective reprotection and simple guest uptake and undergoes a phase transition to the dormant state (Form II) only in response to specific molecular inputs indicates that the dormant state can be selectively reconstructed. This selectivity also supports the presence of a locally defined interaction environment at the deprotected pore surface. Thus, coupling hydrogen‐bond‐based interaction design with structural response enables the dormant state to be selectively re‐formed upon exposure to specific molecular inputs.

### 2.3 Property Changes Accompanying the Phase Transition

#### 2.3.1 Changes in Crystal Shape

The Form I → Form II → Form III SCSC sequence was also clearly accompanied by macroscopic changes in crystal shape. Polarized optical microscopy showed that Form I appeared as a flat and homogeneous plate‐like single crystal, whereas Form II exhibited shortening along the long‐axis direction, followed by further contraction along the long axis and expansion along the short axis in Form III (Figure 5, Table S1). These changes can be understood as macroscopic manifestations of anisotropic lattice deformation occurring at each stage. In particular, the reduction in the long‐axis dimension observed in the first SCSC step (Form I → Form II) was consistent with the shortening of the crystallographic *c* axis and is attributable to the reorganization of the CAHB network from a 1D ladder to a 2D sheet upon the release of the DMSO molecules bonded to *t*BuAH*^+^* (Figure 5). In the second SCSC step (Form II → Form III), the removal of the DMSO molecules protecting the OH groups induced a more extensive framework reorganization, including the rotation and displacement of sulfonate groups (as observed by single‐crystal x‐ray diffraction), accompanied by further contraction along the long axis and elongation along the short axis (Figure 5).

**FIGURE 5:**
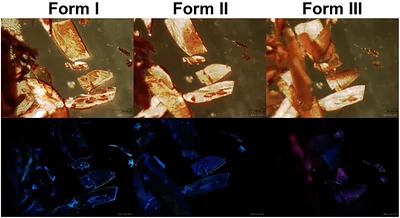
Observation of the macroscopic crystal‐shape changes accompanying the two‐step SCSC transformation. Bright‐field optical microscopy images (top) and optical microscopy images recorded under UV irradiation in the dark (bottom) for Forms I–III.

Importantly, despite these pronounced changes in macroscopic crystal shape, no crystal fracture or significant collapse was observed, and single crystallinity was retained throughout the transformation (Figure 5). Thus, in the present system, molecular‐level rearrangement, changes in the unit‐cell parameters, and macroscopic crystal‐shape changes proceeded continuously via single‐crystal‐to‐single‐crystal transformations. The observed shape change, therefore, reflects not simple thermal deformation or crystal deterioration, but a visually traceable manifestation of hydrogen‐bond network reorganization propagating throughout the crystal lattice. In this sense, the present system provides an SCSC platform in which the relationship between microscopic structural change and macroscopic crystal morphology can be directly monitored [51].

#### 2.3.2 Optical Changes Induced by the Phase Transition

Each activation stage in the SCSC sequence was also clearly distinguished by changes in crystal color. Form I was deep red, Form II was light brown, and Form III was significantly paler, appearing almost white in color (Figure 6a). As a result, these three states could be easily distinguished by the naked eye. Accordingly, the solid‐state ultraviolet–visible (UV–vis) diffuse reflectance spectra showed a stepwise decrease in absorption in the visible region from Form I to Form II and then to Form III, with particularly marked suppression being observed for the long‐wavelength absorption tail (Figure 6b). Additionally, differences among the three states were observed in the solid‐state excitation spectra, emission spectra, average fluorescence lifetimes, and external quantum yields (Figures S13–S17, Table S3), indicating that the optical response changed systematically upon activation [52, 53]. These results reveal that each activation stage corresponds not to a local perturbation but to a discrete bulk‐state change based on SCSC transformation while retaining single crystallinity. Accordingly, the different states can be distinguished not only structurally but also through their optical responses [54], which are readily distinguishable by visual inspection.

**FIGURE 6:**
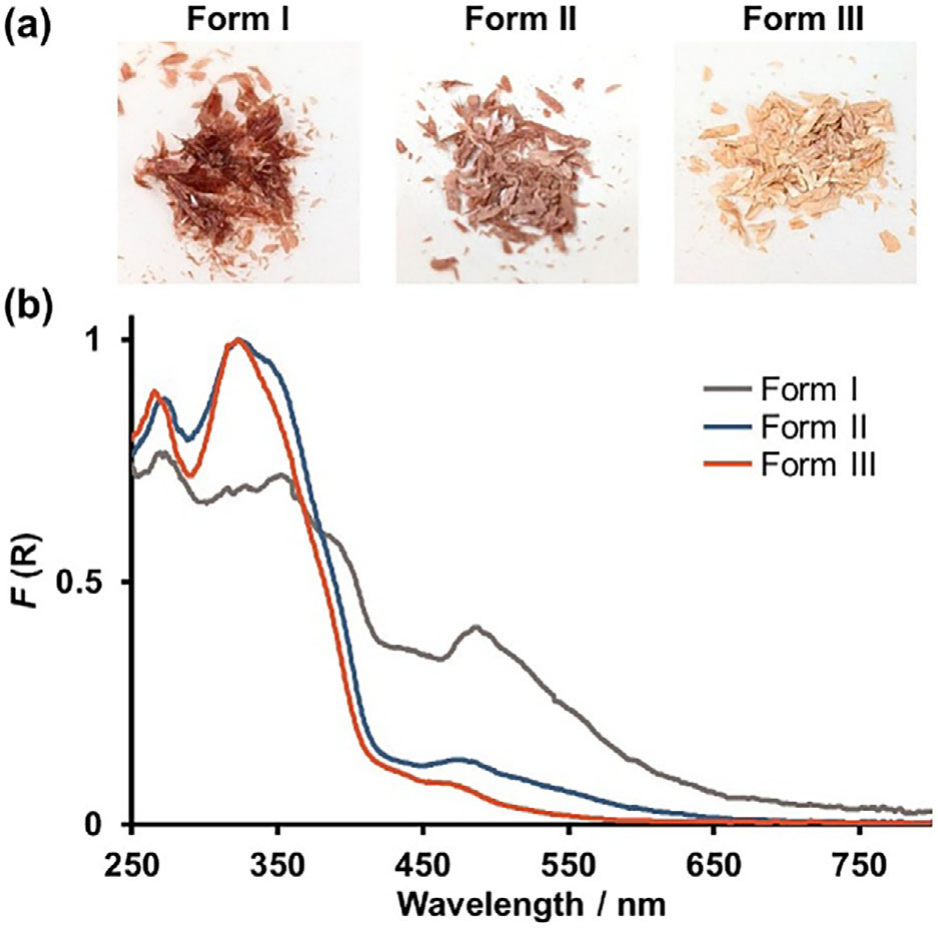
Optical changes accompanying the two‐step SCSC transformation. (a) Photographic images of crystals of Forms I–III. (b) Solid‐state UV–vis diffuse reflectance spectra of Forms I–III, shown as Kubelka–Munk‐transformed data, *F*(*R*).

### 2.4 Gas Adsorption Properties

#### 2.4.1 Emergence of Preferential NH_3_ Adsorption Through the Present Strategy

The key adsorption result of the present system is the emergence of NH_3_‐responsive pore‐surface function upon transformation from the dormant state (Form II) to the active state (Form III). The NH_3_ adsorption isotherm of Form III at 298 K rose steeply from the low‐pressure region, reaching 12.7 mL (STP) g^−1^ at 0.0105 kPa and 22.2 mL (STP) g^−1^ at 0.1286 kPa. In contrast, the extent of CO_2_ adsorption in the same low‐pressure region was extremely small, and minimal adsorption of N_2_, H_2_, and O_2_ was observed over the entire pressure range (Figure 7a). Even near 1 bar, the NH_3_ uptake of Form III reached 89.2 mL (STP) g^−1^, whereas that of CO_2_ was only 2.99 mL (STP) g^−1^, corresponding to an NH_3_/CO_2_ uptake ratio of ∼29.8 (Table S4), revealing the pronounced preference for NH_3_ over CO_2_.

**FIGURE 7:**
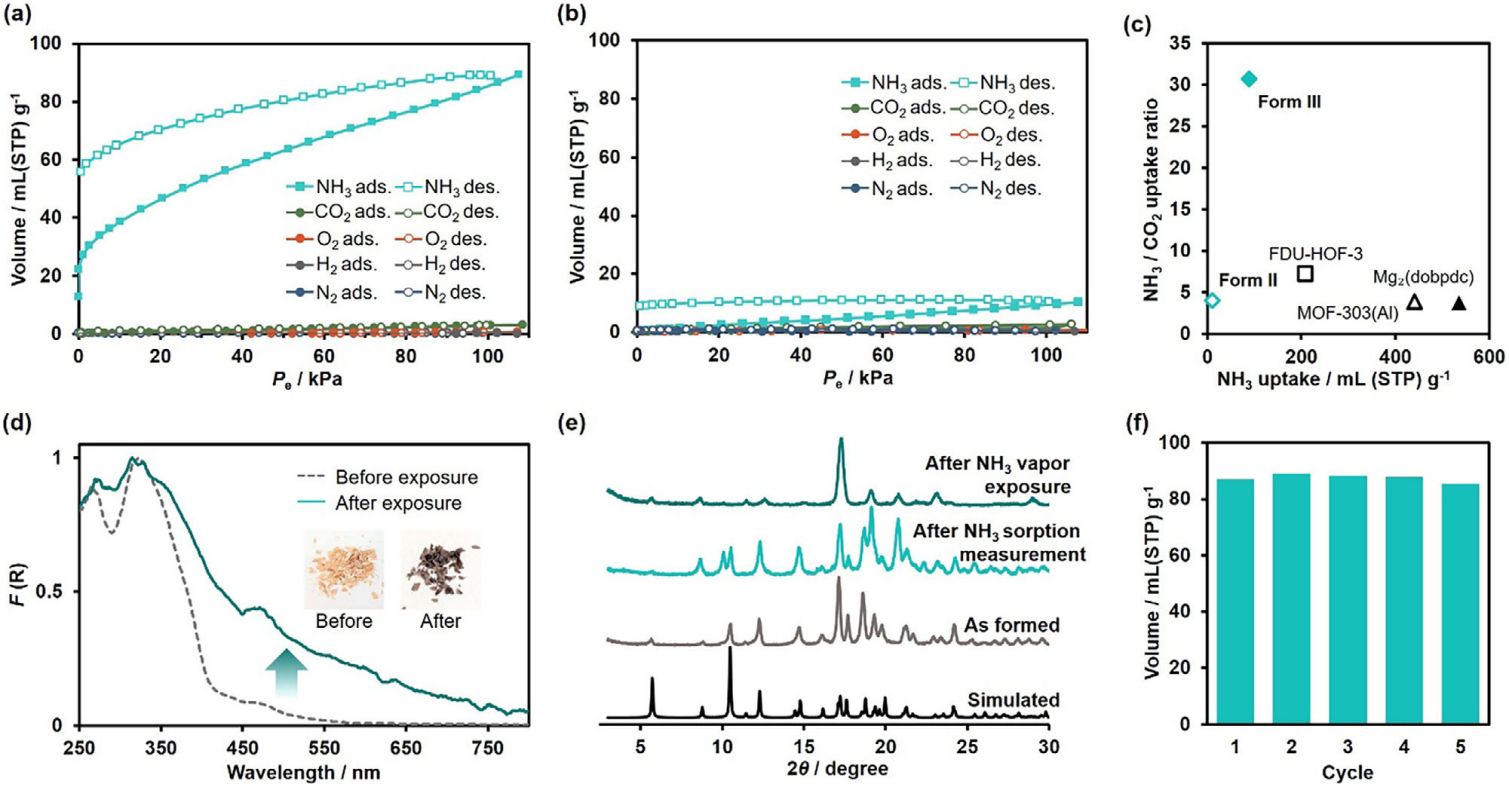
NH_3_‐responsive adsorption properties of the POS. (a) Gas adsorption isotherms of the active state (Form III) for NH_3_, CO_2_, O_2_, H_2_, and N_2_ at 298 K. (b) Gas adsorption isotherms of the dormant state (Form II) for NH_3_, CO_2_, O_2_, H_2_, and N_2_ at 298 K. (c) Comparison of Forms II and III with representative porous materials in terms of NH_3_ uptake and the NH_3_/CO_2_ uptake ratio at 298 K and ∼1 bar [40, 41, 42, 55, 56]. (d) NH_3_ adsorption–desorption cycling performance of Form III. (e) PXRD patterns of Form III immediately following preparation, after the NH_3_ adsorption–desorption measurements, and after exposure to NH_3_ vapor without desorption, together with the simulated pattern. (f) Diffuse reflectance spectra of Form III before and after exposure to aqueous NH_3_ vapor, shown as Kubelka–Munk‐transformed data, *F*(*R*), together with photographic images of the sample before and after exposure.

Such adsorption behavior cannot be explained by pore formation alone. Rather, it is crucial that, through the two‐step structural transformation accompanied by DMSO removal, phenolic OH groups capable of strong interactions with NH_3_ are positioned to function as adsorption sites. The present supramolecular protection–deprotection strategy therefore enables the introduction of phenolic OH groups—which are difficult to install selectively yet can strongly interact with NH_3—_onto the pore surface through controlled structural transformation, yielding a functional material that shows a pronounced adsorption preference for NH_3_.

Temperature‐dependent NH_3_ adsorption isotherms were further measured to estimate the apparent isosteric heat of adsorption, *Q*_st_ (Figure S19). In the reliable uptake region, corresponding approximately to 0.4–0.7 OH‐equivalent, the apparent *Q*_st_ values gradually decreased from approximately 60 to 40–45 kJ mol^−1^ with increasing NH_3_ uptake. The magnitude of these *Q*_st_ values is consistent with appreciable hydrogen‐bonding‐assisted interactions between NH_3_ and the pore surface of Form III, rather than weak nonspecific physisorption alone [36].

The interaction between the deprotected phenolic OH groups and NH_3_ was also supported by IR spectroscopy. After NH_3_ vapor exposure, the broad absorption in the 3500–3000 cm^−1^ region, assigned mainly to phenolic O–H stretching with possible contribution from N‐H stretching, became more pronounced and broadened (Figure S21). An increase in the absorption around 1460 cm^−1^, attributable to N–H bending vibrations of adsorbed NH_3_/NH_3_‐derived species, was also observed. Similar but weaker spectral changes were detected after the NH_3_ adsorption‐desorption measurement, consistent with residual NH_3_ retained after desorption and with the hysteretic NH_3_ adsorption behavior.

#### 2.4.2 Theoretical Analysis of NH_3_ Adsorption Sites in Form III

To further clarify the NH_3_ adsorption sites in Form III, quantum chemical calculations were performed using a cluster model based on the experimentally determined Form III crystal structure. To avoid ambiguity arising from the disorder of the phenolic OH groups in the Form III crystal structure, possible OH‐orientation patterns were first evaluated by quantum chemical calculations, and the most stable pattern was used as a representative framework structure for the subsequent NH_3_ adsorption‐site analysis (Figure S22). Three initial adsorption models were constructed by placing an NH_3_ molecule near the possible interaction sites: exposed phenolic OH groups, SO_3_^−^ moieties, and *t*BuAH*^+^* cations (Figure S23). After geometry optimization, the NH_3_ molecule was commonly stabilized through O–H···N hydrogen‐bonding interactions with the exposed phenolic OH groups. In particular, the models initially placing NH_3_ near the phenolic OH groups and the *t*BuAH*^+^* cations converged to similar optimized structures, in which the dominant interaction was the O–H···N hydrogen bond with the exposed phenolic OH group rather than a direct interaction with the ammonium cation. The model with NH_3_ initially placed near the SO_3_^−^ moiety also showed stabilization involving an O–H···N hydrogen‐bonding interaction. The relative electronic energies, electrostatic potential analysis, and noncovalent interaction plots further supported the favorable interaction environment around the exposed phenolic OH groups (Figure S24 and Table S7). These results indicate that the exposed phenolic OH groups serve as the dominant NH_3_ adsorption sites in Form III, although secondary contributions from the surrounding framework environment, including the ammonium cations, cannot be completely excluded. Because Form II does not possess accessible pores suitable for accommodating NH_3_ molecules, a comparable adsorption‐site calculation for Form II was not performed.

#### 2.4.3 Dormant/Active Control of NH_3_ Adsorption Function by Protection–Deprotection

The dormant/active control of NH_3_ adsorption function through protection–deprotection was clearly demonstrated by the comparison of the gas adsorption behaviors of Forms II and III. In Form II, no distinct crystallographic voids were observed, indicating its essentially nonporous nature; the phenolic OH groups also remained protected by DMSO (Figure S6). Accordingly, the NH_3_ uptake of Form II reached only 10.24 mL (STP) g^−1^ near 1 bar, whereas the CO_2_ uptake was 2.53 mL (STP) g^−1^, corresponding to an NH_3_/CO_2_ uptake ratio of ∼4.05 (Figure 7b, Table S4). Additionally, no pronounced adsorption of N_2_, H_2_, or O_2_ was observed, indicating that Form II generally interacts weakly with various gases, including NH_3_. These characteristics support the assignment of Form II as a dormant state that is adsorption‐inactive prior to functional emergence.

In contrast, Form III exhibited a clear NH_3_ response, and the comparison between Forms II and III directly illustrates reversible switching between the dormant and active adsorption states. Thus, in the present system, structural activation simultaneously induces deprotection of the OH groups and pore formation, enabling reversible switching between an adsorption‐inactive dormant state and an NH_3_‐responsive active state.

#### 2.4.4 Reusability and Regeneration

The exposure of phenolic OH groups on the pore surface enabled reversible NH_3_ adsorption‐desorption and regeneration under relatively mild conditions [57]. Specifically, in the NH_3_ adsorption‐desorption cycling experiments, no marked decrease in uptake was observed even after five cycles (Figure 7d, Table S5). PXRD analysis showed that the diffraction pattern of the sample recovered after the NH_3_ adsorption‐desorption measurement remained consistent with Form III (Figure 7e), indicating that repeated adsorption and desorption did not cause irreversible framework collapse or conversion to Form II. Additionally, TGA of the recovered sample showed a weight‐loss feature below 150°C that was attributable to desorption of NH_3_ (Figure S20), suggesting that the adsorbed NH_3_ can be released under relatively mild conditions.

The pronounced hysteresis observed in the NH_3_ desorption branch is mainly attributed to the slow release of NH_3_ retained in the phenolic‐OH‐lined confined pore environment. In this environment, NH_3_ is stabilized by O‐H···N hydrogen bonding and additional weak interactions, as supported by the IR and DFT analyses. The PXRD pattern after the NH_3_ adsorption‐desorption measurement and the stable uptake over five cycles indicate that this retention is reversible and does not lead to permanent degradation of the crystalline framework. This behavior contrasts with adsorption via strong coordination to metal centers and indicates that the present system combines sufficient NH_3_ affinity with relatively mild regeneration, demonstrating its potential as a reusable NH_3_ adsorbent.

#### 2.4.5 Visible Response Upon NH_3_ Adsorption

NH_3_ adsorption by Form III was also directly observed as a visible response. Upon exposure to vapor from a 35% aqueous NH_3_ solution, the sample changed color within 5 min, accompanied by a large change in the absorption spectrum (Figure 7f). This reveals that Form III can adsorb NH_3_ not only under high‐vacuum gas adsorption conditions but also under milder vapor‐exposure conditions. Furthermore, TGA of the exposed sample showed mass changes consistent with the uptake of NH_3_‐containing guest molecules (Figure S20), whereas PXRD indicated that the diffraction pattern after NH_3_ vapor exposure remained consistent with Form III (Figure 7e). As a control experiment, Form III was exposed to water vapor under otherwise similar conditions. In contrast to the NH_3_‐vapor‐exposed sample, no apparent color change was observed after water‐vapor exposure. In addition, TGA of the water‐vapor‐exposed sample showed no appreciable additional weight‐loss feature corresponding to guest uptake (Figure S20). These results indicate that the visible color change and mass change observed after exposure to vapor from the aqueous NH_3_ solution are not caused by water vapor alone but are associated with NH_3_ adsorption.

Thus, in contrast to DMSO‐induced reprotection, which involves structural transformation, NH_3_ uptake occurred while retaining the Form III crystalline framework, and this process manifested as a clear color change. Accordingly, Form III functions as a responsive adsorbent that captures NH_3_ while retaining crystallinity and visually reports this process through an optical response. Rather than aiming at a maximum uptake capacity, the present system is distinguished by the large contrast between NH_3_ and CO_2_ uptake and by protection–deprotection‐based switching of NH_3_ adsorption (Figure 7c). Thus, the key feature of this system is not its uptake capacity alone, but the temporal control over the emergence of NH_3_‐responsive pore‐surface functionality.

## 3 Conclusion

In conclusion, this study developed a supramolecular protection–deprotection strategy in a crystalline POS that temporally separated framework construction from the emergence of pore‐surface functionality. During crystallization, phenolic OH groups were protected by hydrogen bonding with DMSO, enabling the formation of a crystalline framework based on charge‐assisted hydrogen bonds. Stepwise removal of DMSO subsequently induced a two‐step single‐crystal‐to‐single‐crystal phase transition, affording a dormant state (Form II), in which the OH groups remained protected, and an active state (Form III), in which the OH groups were exposed on the pore surface together with the emergence of porosity. The dormant state was re‐formed upon exposure to DMSO or DMA vapor, whereas other solvents did not induce the same structural response. In contrast, the active state exhibited markedly enhanced NH_3_ uptake relative to the dormant state, together with the preferential adsorption of NH_3_ over CO_2_, N_2_, H_2_, and O_2_ under the present conditions. These findings show that supramolecular protection–deprotection represents a key strategy for the temporal installation of highly interactive functional groups within a crystalline porous framework and for the reversible switching of the pore surface between dormant and active states. This strategy may be extendable to other highly interactive functional groups by appropriate selection of supramolecular protecting partners, thereby broadening the scope of switchable pore‐surface engineering in crystalline porous materials.

## Author Contributions

**Kazuki Shiga**: writing – original draft, formal analysis, investigation, validation, methodology, software, visualization, conceptualization, data curation. **Ryota Yamazaki**: investigation, formal analysis, writing – review and editing, validation, visualization. **Hajime Shigemitsu**: methodology, software, investigation, formal analysis, visualization, writing – review and editing. **Yuuya Nagata**: methodology, formal analysis, writing – review and editing, supervision, software, investigation. **Norimitsu Tohnai**: conceptualization, methodology, writing – original draft, supervision, validation, funding acquisition, project administration, data curation.

## Conflicts of Interest

The authors declare no conflicts of interest.

## Supporting information




**Supporting File**: anie73973‐sup‐0001‐SuppMat.docx.

## Data Availability

The data that supports the findings of this study are available in the Supporting Information of this article.
