# Supplementary material for: Supramolecular Protection–Deprotection for Switching Pore‐Surface Functionality in a Crystalline Porous Organic Salt
Source: Angew Chem Int Ed Engl. 2026 Aug 5;65(40):e1547062. doi: 10.1002/anie.1547062 (PMC13618268; doi:10.1002/anie.1547062)
Supplement: Supplementary file 1 [file ANIE-65-e1547062-s001.docx]

Supporting Information

**Supramolecular Protection–Deprotection for Switching Pore-Surface Functionality in a Crystalline Porous Organic Salt**

Kazuki Shiga^[a]^, Ryota Yamazaki^[a]^, Hajime Shigemitsu^[b]^, Yuuya Nagata^[b]^, and Norimitsu Tohnai*^[a]^

[a]	K. Shiga, R. Yamazaki, Prof. Dr. N. Tohnai*
Department of Applied Chemistry
Graduate School of Engineering, The University of Osaka
2-1 Yamadaoka, Suita, Osaka 565-0871, Japan
E-mail: [tohnai@chem.eng.osaka-u.ac.jp](mailto:tohnai@chem.eng.osaka-u.ac.jp)

[b]	Dr. H. Shigemitsu, Dr. Y. Nagata
Research Center for Macromolecules and Biomaterials (RCMB)
National Institute for Materials Science (NIMS)
1-2-1 Sengen, Tsukuba, Ibaraki 305-0047, Japan

1. General Information
2. Materials and Instrumentation
3. Synthesis of HQDPS
4. Preparation of HQDPS/*t*BuA Salt and Crystallization of Forms I and the Control Crystal
5. Vapor Exposure Experiments
6. Single-Crystal X-ray Diffraction
7. Powder X-ray Diffraction
8. Thermogravimetric Analysis
9. Gas Sorption Measurements
10. IR Spectroscopic Analysis after NH_3_ Uptake
11. Diffuse Reflectance and Photoluminescence Measurements
12. Supplementary Discussion on Morphological and Optical Changes Accompanying the SCSC Transformation
13. Supplementary Figures and Tables
14. Crystallographic Data
15. Computational Methods
16. References

**1. General Information**

All reagents and solvents were obtained from commercial suppliers and used as received unless otherwise noted. Hydroquinone diphenyl sulfonic acid (HQDPS) was synthesized according to the procedure described below. Bulk crystalline samples and single crystals of Forms I–III were prepared as described in the corresponding sections. All sample names, including Forms I–III, are used consistently throughout this Supporting Information and the main text.

**2. Materials and Instrumentation**

Single-crystal X-ray diffraction (SCXRD) data were collected using a Rigaku XtaLAB PRO diffractometer equipped with Cu Kα radiation. Powder X-ray diffraction (PXRD) patterns were recorded using a Rigaku Ultima IV diffractometer with Cu Kα radiation. Thermogravimetric (TG) analyses were performed using a Rigaku Thermo Plus EVO2 TG8121 instrument under a nitrogen flow. ^1^H NMR spectra were recorded on a JEOL ECZ400 series spectrometer using appropriate deuterated solvents. Steady-state photoluminescence spectra and excitation spectra in both the solid and solution states, as well as solid-state diffuse reflectance spectra and external quantum yield measurements, were carried out on a JASCO FP-8500 spectrofluorometer. Solution UV–vis absorption spectra were measured on a JASCO V-770 spectrophotometer. Fluorescence lifetime measurements were performed using the instrument described in the corresponding section. Diffuse reflectance data are shown as Kubelka–Munk-transformed spectra, *F*(*R*). Gas sorption measurements were performed using BELSORP-max X and BELSORP MAX instruments (MicrotracBEL Corp., Osaka, Japan). Unless otherwise noted, the samples were pretreated before measurement under the conditions described in the corresponding section. IR spectra were recorded using a JASCO FT/IR-4200 Fourier-transform infrared spectrometer equipped with a diamond attenuated total reflectance (ATR) accessory.

**3. Synthesis of HQDPS**

HQDPS was synthesized according to the procedure described below, and the intermediates and product were characterized by NMR spectroscopy (**Figures S1–S5**).

**3.1 Dineopentyl 2',5'-dimethoxy-[1,1':4',1''-terphenyl]-4,4''-disulfonate (DMTPDS-Np)**

A 200 mL round-bottom flask was charged with 1,4-dibromo-2,5-dimethoxybenzene (1.00 g, 3.38 mmol, 1.0 eq.), Neopentyl 4-(4,4,5,5-tetramethyl-1,3,2-dioxaborolan-2-yl)benzenesulfonate (2.98 g, 8.45 mmol, 2.5 eq.), Pd(PPh_3_)_4_ (0.394 g, 0.342 mmol, 0.10 eq.), sodium carbonate (3.58 g, 33.8 mmol, 10 eq.), and Aliquat 336 (0.169 g, 0.418 mmol, 0.12 eq.). After replacement of the atmosphere with nitrogen, nitrogen-bubbled toluene (55 mL), ethanol (18 mL), and water (22 mL) were added successively, and the reaction mixture was refluxed at 90 °C overnight. After completion of the reaction, toluene and ethanol were removed under reduced pressure, and ethyl acetate (100 mL) was added. The mixture was filtered. The residue was dissolved in chloroform (100 mL), washed with water (3 × 30 mL), dried over MgSO_4_, filtered, and concentrated under reduced pressure. The crude product was purified by flash column chromatography (CHCl_3_) to afford DMTPDS-Np as a white solid (1.52 g, 76.5%).

^1^H NMR (400 MHz, CDCl_3_) *δ* 7.96 (d, *J* = 8.0 Hz, 4H), 7.76 (d, *J* = 8.0 Hz, 4H), 6.99 (s, 2H), 3.83 (s, 6H), 3.77 (s, 4H), 0.95 (s, 18H). (**Figure S1**)

^13^C NMR (400 MHz, CDCl_3_) *δ* 150.7, 143.5, 134.7, 130.1, 129.4, 127.7, 114.5, 79.7, 77.0, 76.7, 56.4, 31.7, 26.0. (**Figure S2**)

**Figure S1**. ^1^H NMR spectrum (400 MHz, CDCl_3_) of DMTPDS-Np.

**Figure S2**. ^13^C NMR spectrum (400 MHz, CDCl_3_) of DMTPDS-Np.

**3.2 bis(neopentyl) 2',5'-dioxo-2',5'-dihydro-[1,1':4',1''-terphenyl]-4,4''-disulfonate (BQDPS-Np)**

A 20 mL round-bottom flask was charged with ceric ammonium nitrate (984 mg, 1.79 mmol, 7.4 eq.) and water (3.4 mL), and the mixture was stirred at 26 °C. A solution of DMTPDS-Np (148 mg, 0.251 mmol, 1.0 eq.) in THF (7.0 mL) was added dropwise. After the addition, water (5 mL) was added, and the crude product was collected by filtration. After drying, the crude solid was purified by silica gel column chromatography (CHCl_3_) to afford BQDPS-Np as a yellow solid (105 mg, 74.8%).

^1^H NMR (400 MHz, CDCl_3_) *δ* 8.01 (d, *J* = 8.0 Hz, 4H), 7.73 (d, *J* = 8.0 Hz, 4H), 7.07 (s, 2H), 3.76 (s, 6H), 0.94 (s, 18H). (**Figure S3**)

^13^C NMR (400 MHz, CDCl_3_) *δ* 185.5, 144.1, 137.7, 137.2, 134.3, 130.1, 128.0, 80.1, 31.8, 26.0. (**Figure S4**)

**Figure S3**. ^1^H NMR spectrum (400 MHz, CDCl_3_) of BQDPS-Np.

**Figure S4**. ^13^C NMR spectrum (400 MHz, CDCl_3_) of BQDPS-Np.

|  |  |
| --- | --- |
| 12.5372 | -0.000992251 |
| 12.5361 | -0.000268934 |
| 12.535 | -0.000270882 |
| 12.5338 | -0.000352161 |
| 12.5327 | -0.000742877 |
| 12.5315 | -0.000939713 |
| 12.5304 | -0.000459629 |
| 12.5292 | -0.000347105 |
| 12.5281 | -0.000688281 |
| 12.5269 | 9.17474e-06 |
| 12.5258 | -0.000613554 |
| 12.5247 | -3.50451e-06 |
| 12.5235 | -0.000250647 |
| 12.5224 | -0.000750325 |
| 12.5212 | -0.000413445 |
| 12.5201 | -0.000326434 |
| 12.5189 | -0.000747089 |
| 12.5178 | -0.000349006 |
| 12.5166 | -0.000261544 |
| 12.5155 | -0.000517087 |
| 12.5144 | -0.000563491 |
| 12.5132 | -0.00100583 |
| 12.5121 | -0.000622077 |
| 12.5109 | -0.000383189 |
| 12.5098 | -0.000649149 |
| 12.5086 | -0.000613191 |
| 12.5075 | -0.00100967 |
| 12.5063 | -0.000285919 |
| 12.5052 | -0.000148584 |
| 12.5041 | -0.000354476 |
| 12.5029 | -0.000277713 |
| 12.5018 | -0.000992338 |
| 12.5006 | -0.000774677 |
| 12.4995 | -0.000547848 |
| 12.4983 | -0.000305576 |
| 12.4972 | -0.00107548 |
| 12.4961 | -0.000781457 |
| 12.4949 | -0.00073987 |
| 12.4938 | -0.000709082 |
| 12.4926 | -0.000471069 |
| 12.4915 | 6.07341e-05 |
| 12.4903 | -0.000355541 |
| 12.4892 | -0.000447137 |
| 12.488 | -0.000667759 |
| 12.4869 | -0.0010178 |
| 12.4858 | -0.000257549 |
| 12.4846 | -0.000315411 |
| 12.4835 | -0.000322826 |
| 12.4823 | -0.000698483 |
| 12.4812 | -0.000823098 |
| 12.48 | 0.000192863 |
| 12.4789 | -0.000273182 |
| 12.4777 | -0.000345136 |
| 12.4766 | -0.000525664 |
| 12.4755 | -0.000423291 |
| 12.4743 | -0.00101194 |
| 12.4732 | -0.000379069 |
| 12.472 | -0.000531716 |
| 12.4709 | -0.000172546 |
| 12.4697 | -0.000647098 |
| 12.4686 | -0.000889256 |
| 12.4674 | -0.000915035 |
| 12.4663 | -0.000617697 |
| 12.4652 | -0.00123153 |
| 12.464 | -0.00136383 |
| 12.4629 | -0.000484746 |
| 12.4617 | -0.000758 |
| 12.4606 | -0.00101251 |
| 12.4594 | -0.0015502 |
| 12.4583 | -0.00129204 |
| 12.4572 | -0.00048476 |
| 12.456 | -0.000604159 |
| 12.4549 | -0.00071278 |
| 12.4537 | -0.00127972 |
| 12.4526 | -0.00112342 |
| 12.4514 | -0.000774738 |
| 12.4503 | -0.000697242 |
| 12.4491 | -0.000449333 |
| 12.448 | -0.000739275 |
| 12.4469 | -0.00074377 |
| 12.4457 | -0.000156239 |
| 12.4446 | -0.000453428 |
| 12.4434 | -0.000247064 |
| 12.4423 | -0.00111396 |
| 12.4411 | -0.00109974 |
| 12.44 | -0.000302401 |
| 12.4388 | -0.000498864 |
| 12.4377 | -0.000686393 |
| 12.4366 | -0.000471969 |
| 12.4354 | -0.000348704 |
| 12.4343 | -0.000186517 |
| 12.4331 | -0.000509728 |
| 12.432 | -0.000637699 |
| 12.4308 | -0.000795146 |
| 12.4297 | -0.000408044 |
| 12.4285 | -0.000685921 |
| 12.4274 | -0.000526403 |
| 12.4263 | -0.000259536 |
| 12.4251 | -0.000551374 |
| 12.424 | -0.000629443 |
| 12.4228 | -0.000243808 |
| 12.4217 | -0.000727342 |
| 12.4205 | -0.000372655 |
| 12.4194 | -0.000504159 |
| 12.4183 | -0.000458801 |
| 12.4171 | -0.00100156 |
| 12.416 | -0.000542427 |
| 12.4148 | -0.00138806 |
| 12.4137 | -0.000535154 |
| 12.4125 | -0.000690267 |
| 12.4114 | -0.000202399 |
| 12.4102 | -0.000679759 |
| 12.4091 | -0.000719425 |
| 12.408 | -0.000308019 |
| 12.4068 | -0.000170145 |
| 12.4057 | -0.000623583 |
| 12.4045 | -0.00062638 |
| 12.4034 | -0.00045334 |
| 12.4022 | -0.0010045 |
| 12.4011 | -0.00113705 |
| 12.3999 | -0.000903745 |
| 12.3988 | -0.000956325 |
| 12.3977 | -0.000656079 |
| 12.3965 | -0.000725547 |
| 12.3954 | -0.000980308 |
| 12.3942 | -0.000387098 |
| 12.3931 | -0.000560273 |
| 12.3919 | -0.000590123 |
| 12.3908 | -0.000411338 |
| 12.3896 | -0.000386001 |
| 12.3885 | -0.000440744 |
| 12.3874 | -0.000833874 |
| 12.3862 | -0.000652282 |
| 12.3851 | -0.000262752 |
| 12.3839 | -0.000149804 |
| 12.3828 | -0.000656039 |
| 12.3816 | -0.00117422 |
| 12.3805 | -0.000582549 |
| 12.3794 | -0.000504384 |
| 12.3782 | -0.00062125 |
| 12.3771 | -0.000215261 |
| 12.3759 | -0.00103982 |
| 12.3748 | -0.000998309 |
| 12.3736 | -0.000716628 |
| 12.3725 | -0.00064022 |
| 12.3713 | -0.000184327 |
| 12.3702 | -0.000617389 |
| 12.3691 | -0.000152452 |
| 12.3679 | -0.000693657 |
| 12.3668 | -0.000178085 |
| 12.3656 | -0.000497689 |
| 12.3645 | -0.000900332 |
| 12.3633 | -0.000933842 |
| 12.3622 | -0.000198381 |
| 12.361 | -0.000803387 |
| 12.3599 | -0.000658695 |
| 12.3588 | -0.0002199 |
| 12.3576 | -0.00109364 |
| 12.3565 | -0.000397546 |
| 12.3553 | -0.000106077 |
| 12.3542 | -0.00052623 |
| 12.353 | -0.000461991 |
| 12.3519 | -0.00102246 |
| 12.3507 | -0.000456112 |
| 12.3496 | -0.000693325 |
| 12.3485 | 0.000441194 |
| 12.3473 | -6.09959e-05 |
| 12.3462 | -0.000505154 |
| 12.345 | -0.000629193 |
| 12.3439 | -0.000915401 |
| 12.3427 | -0.000874185 |
| 12.3416 | -0.000269941 |
| 12.3405 | -0.00059847 |
| 12.3393 | -0.000167356 |
| 12.3382 | -0.000321476 |
| 12.337 | -0.000916456 |
| 12.3359 | -0.00113182 |
| 12.3347 | -0.00176346 |
| 12.3336 | -0.00110307 |
| 12.3324 | -0.000580491 |
| 12.3313 | -0.0011459 |
| 12.3302 | -0.000731512 |
| 12.329 | -0.000535129 |
| 12.3279 | -0.000250325 |
| 12.3267 | -0.000928582 |
| 12.3256 | -0.00044889 |
| 12.3244 | -0.00038633 |
| 12.3233 | -0.0006241 |
| 12.3221 | -0.000941024 |
| 12.321 | -0.00022518 |
| 12.3199 | -0.000512504 |
| 12.3187 | -0.000779416 |
| 12.3176 | -0.000880221 |
| 12.3164 | -0.000598815 |
| 12.3153 | -0.000320878 |
| 12.3141 | -0.000591796 |
| 12.313 | -0.000814857 |
| 12.3118 | -0.000289253 |
| 12.3107 | -0.000600457 |
| 12.3096 | -0.000373129 |

**3.3 2',5'-dihydroxy-[1,1':4',1''-terphenyl]-4,4''-disulfonic acid (HQDPS)**

BQDPS-Np (118 mg, 1.0 eq.) was placed in a 50 mL round-bottom flask, and 14.1 mL trifluoroacetic acid was added. The reaction mixture was refluxed at 90 °C for 21 h. After completion of the reaction, the mixture was concentrated under reduced pressure and dried. Ethyl acetate (1.0 mL) was then added, and the mixture was heated at 90 °C. After the treatment, the resulting solid was collected by filtration and dried to afford HQDPS as a white solid (74.2 mg, 84%).

^1^H NMR (400 MHz, DMSO-*d*_6_) *δ* 7.61 (d, *J* = 8.0 Hz, 4H), 7.59 (d, *J* = 8.0 Hz, 4H), 6.85 (s, 2H). (**Figure S5**)

**Figure S5**. ^1^H NMR spectrum (400 MHz, DMSO-*d*_6_) of HQDPS.

**4. Preparation of HQDPS/*t*BuA Salt and Crystallization of Forms I and Control Crystal**

**4.1 Preparation of the HQDPS/*t*BuA salt**

HQDPS and tert-butylamine (*t*BuA) were combined in methanol at 298 K using 2.5 eq. of *t*BuA with respect to HQDPS. After salt formation, methanol was removed under reduced pressure. The resulting solid was washed with diethyl ether to remove excess amine and then dried to afford the HQDPS/*t*BuA salt with a 1:2 stoichiometry.

**4.2 Preparation of Form I**

The crude HQDPS/*t*BuA salt (10 mg) was dissolved in DMSO (200 μL), and Form I was obtained by vapor diffusion of ethyl acetate into the resulting solution at 298 K for ca. 12 h.

**4.3 Preparation of the control crystal from MeOH**

For the control experiment, the crude HQDPS/*t*BuA salt (10 mg) was dissolved in methanol (200 μL), and crystals were obtained by vapor diffusion of diethyl ether into the solution at 298 K for ca. 12 h.

**4.4 Preparation of Form II**

Form II was obtained by heating Form I at 100 °C for 1 h.

**4.5 Preparation of Form III**

Form III was obtained by heating Form II at 160 °C for 1 h.

**5. Vapor Exposure Experiments**

Vapor-exposure experiments were conducted using a nested-vial setup consisting of a small inner sample tube and a larger outer sample tube. The inner tube was a Maruemu sample tube (No. 1, 4 mL, 16.5 mm body diameter, 40 mm length), and the outer tube was a Maruemu sample tube (No. 6, 30 mL, 20.8 mm inner diameter, 30 mm body diameter, 63 mm length).

**5.1. DMSO vapor exposure**

Crystals of Form III (10 mg) were placed in the inner sample tube, which was then inserted into the outer sample tube. DMSO (1 mL) was added to the outer tube without direct contact with the crystals, and the outer tube was sealed. The sample was kept at 298 K for 24 h. After the exposure, the resulting sample was subjected to PXRD and TG measurements.

**5.2. Reversible protection–deprotection cycling**

Reversible switching between Forms II and III was examined by alternating DMSO-vapor exposure and thermal treatment. Exposure of Form III to DMSO vapor under the conditions described above regenerated Form II, whereas subsequent heating converted Form II back to Form III.

**5.3. Exposure to other solvent vapors**

Crystals of Form III (10 mg) were exposed, under conditions analogous to those used for DMSO-vapor exposure, to vapors of DMA, DMF, acetone, and 2-propanol. Specifically, the crystals were placed in the inner sample tube, which was inserted into the outer sample tube, and 1 mL of each solvent was added to the outer tube without direct contact with the crystals. The outer tube was sealed, and the sample was kept at 298 K for 24 h. The resulting samples were analyzed by PXRD to evaluate whether structural conversion to Form II had occurred.

**5.4. NH_3_ vapor exposure**

For evaluation of the visible response, crystals of Form III (10 mg) were placed in the inner sample tube, which was inserted into the outer sample tube. Aqueous ammonia solution (35%, 1 mL) was added to the outer tube without direct contact with the crystals, and the outer tube was sealed. The sample was exposed to ammonia vapor at 298 K for less than 5 min. The resulting sample was analyzed by diffuse reflectance spectroscopy, PXRD, and TG measurements.

As a control experiment to evaluate the influence of water vapor, Form III was exposed to water vapor under otherwise similar conditions. Crystals of Form III (10 mg) were placed in the inner sample tube, and deionized water (1 mL) was added to the outer tube without direct contact with the crystals. The outer tube was sealed, and the sample was kept at 298 K for 5 min. The resulting sample was analyzed by visual inspection and TG measurement.

**6. Single-Crystal X-ray Diffraction**

Single-crystal X-ray diffraction (SCXRD) data were collected using a Rigaku XtaLAB PRO diffractometer equipped with Cu Kα radiation (λ = 1.54187 Å). The obtained diffraction intensity data were analyzed using Olex2.^[60]^ The structures were solved by intrinsic phasing with SHELXT^[61]^ and refined by full-matrix least-squares on *F*^2^ using SHELXL.^[62]^ Non-hydrogen atoms were refined anisotropically. Hydrogen atoms were placed in geometrically calculated positions and refined using a riding model. For porous structures containing highly disordered solvent molecules, solvent contributions were treated using PLATON/SQUEEZE where appropriate. ^[63]^

**6.1. Structure determination of Forms I–III**

SCXRD analysis was carried out for Forms I–III to determine the structural changes accompanying the two-step single-crystal-to-single-crystal (SCSC) transformation. Form I corresponds to the DMSO-protected phase obtained by recrystallization from DMSO, Form II to the intermediate phase obtained after the first thermal treatment, and Form III to the deprotected phase obtained after the second thermal treatment. Structural parameters for each phase are summarized in **Table S1**, and the corresponding crystal structures are shown in **Figures S6** and **S7**.

**6.2. Crystallographic interpretation of the two-step transformation**

The structural analyses showed that the multiple DMSO molecules present in Form I were crystallographically nonequivalent and exhibited distinct behavior depending on their local binding environments. In the first SCSC step, DMSO molecules hydrogen-bonded to the ammonium cations were selectively removed to afford Form II, while the DMSO molecules hydrogen-bonded to the phenolic OH groups were retained. In the second SCSC step, the remaining DMSO molecules protecting the OH groups were removed, affording Form III. These structural changes were accompanied by reorganization of the charge-assisted hydrogen-bonding (CAHB) network from a one-dimensional ladder in Form I to a two-dimensional sheet in Form II and finally to a three-dimensional network in Form III.

**6.3. Structural analysis of the control crystal obtained from MeOH**

For comparison, the crystal obtained by recrystallization of the HQDPS/*t*BuA salt from methanol was also analyzed by SCXRD (**Figure S8** and **Table S2**). In this control structure, the OH groups directly participated in the hydrogen-bonding network instead of being supramolecularly protected by DMSO, providing a reference for discussing the role of DMSO in the formation of Form I.

**6.4. Structural analysis of TPDS-OMe_2_/*t*BuA control crystals**

To further examine whether the OH groups supramolecularly protected by DMSO in HQDPS/*t*BuA are placed in a state structurally analogous to that of covalently protected phenol units, the methoxy-substituted analogue 2',5'-dimethoxy-[1,1':4',1''-terphenyl]-4,4''-disulfonic acid (TPDS-OMe_2_)/*t*BuA was recrystallized under conditions similar to those used for HQDPS/*t*BuA. As a result, two crystalline polymorphs, denoted TPDS-OMe_2_/*t*BuA Form I and Form II, were obtained (**Figure S9**). Both structures were closely related to HQDPS/*t*BuA Form I. TPDS-OMe_2_/*t*BuA Form I contained no solvent molecules other than DMSO, whereas TPDS-OMe_2_/*t*BuA Form II incorporated ethyl acetate molecules derived from the antisolvent between the sulfonate-bearing molecular units.

In both TPDS-OMe_2_/*t*BuA polymorphs, DMSO molecules were hydrogen-bonded to the *t*BuAH+ cations, and the sulfonate–ammonium charge-assisted hydrogen bonds formed a one-dimensional ladder-type network composed of two parallel one-dimensional hydrogen-bonded chains. This network motif closely resembles that observed in HQDPS/*t*BuA Form I. In TPDS-OMe_2_, the phenolic OH groups are replaced by methoxy groups; therefore, the corresponding sites are covalently masked rather than supramolecularly protected by DMSO. Nevertheless, the local structure around the methoxy-substituted terphenyl unit corresponded well to that around the DMSO-protected OH groups in HQDPS/*t*BuA Form I. This comparison indicates that the DMSO-protected state in HQDPS/*t*BuA provides an environment structurally analogous to that of a covalently O-methyl-masked state. Therefore, these control structures support the interpretation that DMSO functions as a supramolecular protecting molecule for the phenolic OH groups in HQDPS/*t*BuA Form I.

**6.5. Structural analysis of DMA-recrystallized HQDPS/*t*BuA**

To further examine the role of DMA in the reprotection process, the HQDPS/*t*BuA salt was recrystallized from DMA. Single-crystal X-ray diffraction analysis revealed that the obtained crystal structure was closely related to HQDPS/*t*BuA Form II (**Figure S12**). In this structure, the phenolic OH groups were hydrogen-bonded to DMA molecules, indicating that DMA can supramolecularly protect the phenolic OH groups in a manner analogous to the DMSO protection observed in Form II.

The overall charge-assisted hydrogen-bonding network was also closely related to that of Form II. The sulfonate–ammonium interactions formed a two-dimensional network, and these two-dimensional sheets were stacked to construct the crystal structure. Thus, the DMA-recrystallized structure supports the interpretation that DMA has sufficient hydrogen-bond-accepting ability to reprotect the exposed phenolic OH groups and stabilize a Form II-like dormant state.

Notably, unlike HQDPS/*t*BuA Form I obtained from DMSO, no clear hydrogen bond between the *t*BuAH^+^ cation and DMA was observed in the DMA-recrystallized structure. This difference indicates that DMA can protect the phenolic OH groups but does not reproduce all of the local interactions observed for DMSO in Form I. Therefore, the structural comparison suggests that the ability of DMA to induce reprotection mainly arises from its hydrogen-bond-accepting interaction with the phenolic OH groups, whereas the additional stabilization around the *t*BuAH^+^ cations observed in the DMSO-containing Form I is more specific to DMSO.

**6.6. Evaluation of pore aperture and void fraction**

The apparent pore aperture of Form III was evaluated from the crystallographic dataset. The channel aperture was estimated from the crystal structure using the crystallographic dimensions as a scale and was determined to be ca. 6.7 × 4.0 Å. The solvent-accessible void volume was calculated using PLATON to be 294.8 Å^3^ per unit cell. Considering the unit-cell volume of Form III (1702.3 Å^3^), this value corresponds to a void fraction of 17.3%.

It should be noted that this aperture represents an apparent opening estimated from the static crystallographic model. In Form III, the central benzene ring of the terphenyl moiety is crystallographically disordered, giving two possible local pore shapes **(Figure S11**). One orientation provides the open channel described above, whereas the other orientation protrudes into the channel and narrows the local bottleneck aperture to approximately 1.8 Å. Therefore, although Form III possesses crystallographic one-dimensional channels and a measurable void fraction, the effective diffusion pathway can be partially restricted by local pore-shape disorder. This structural feature provides a reasonable explanation for why the CO_2_ uptake of Form III is lower than might be expected from the apparent crystallographic aperture and void fraction.

**7. Powder X-ray Diffraction**

Powder X-ray diffraction (PXRD) patterns were recorded using a Rigaku Ultima IV diffractometer with Cu Kα radiation at 298 K unless otherwise noted. Bulk crystals obtained by recrystallization, thermal treatment, vapor exposure, or gas sorption measurements were placed on a silicon plate for measurement. When solvent-containing samples were measured, excess surface solvent was removed as much as possible with filter paper before measurement. Data were collected over the 2*θ* range of 3–30° with a step width of 0.02° and a scanning speed of 3° min^−1^. Simulated PXRD patterns were generated from the corresponding single-crystal structures.

**7.1. PXRD analysis of Forms I–III**

PXRD measurements were used to evaluate whether the phase transformations observed by SCXRD occurred throughout the bulk samples (**Figure 3a**). The diffraction patterns of Forms I–III were clearly distinct from one another and were in good agreement with the corresponding simulated patterns, supporting bulk conversion to single new phases at each activation stage.

**7.2. PXRD analysis after DMSO-vapor exposure and repeated cycling**

PXRD measurements were also performed for samples obtained after DMSO-vapor exposure of Form III and after repeated protection–deprotection cycling (**Figure 4a**). The pattern after DMSO-vapor exposure was consistent with that of Form II, supporting vapor-induced reconstruction of the dormant state. In the repeated cycling experiments, the characteristic diffraction peaks of Forms II and III were retained after each cycle, indicating reversible interconversion without marked loss of crystallinity.

**7.3. PXRD analysis after exposure to other solvent vapors**

PXRD was used to evaluate structural responses of Form III to vapors of DMA, DMF, acetone, and 2-propanol (**Figure 4d**). Under the present conditions, DMA induced conversion to a Form II-like phase, whereas the other tested solvents did not show the same pronounced structural response.

**7.4. PXRD analysis after NH_3_ exposure and after NH_3_ adsorption–desorption measurement**

To examine the structural stability of Form III toward NH_3_, PXRD measurements were carried out for samples after exposure to NH_3_ vapor and for samples recovered after the NH_3_ adsorption–desorption measurement (**Figure 7e**). In both cases, the diffraction patterns remained consistent with Form III. The main diffraction peaks corresponding to Form III were retained, and no additional set of peaks assignable to Form II or another crystalline phase was observed. The differences observed among the patterns were mainly in relative peak intensities, which can arise from preferred orientation and sample-packing effects of the plate-like crystals. These results support that NH_3_ uptake proceeds while retaining the Form III crystalline framework, without inducing the reverse phase transition to Form II observed upon DMSO reprotection.

**8. Thermogravimetric Analysis**

Thermogravimetric (TG) analyses were performed using a Rigaku Thermo Plus EVO2 TG8121 instrument. Measurements were carried out under a nitrogen flow of 100 mL min^-1^. Samples (typically >1.2 mg) were heated from 298 K to 400 °C at a heating rate of 3 °C min^-1^. Alumina pre-dried to remove adsorbed moisture was used as the reference material. TG measurements were conducted for Forms I–III, the DMSO-vapor-exposed sample, the NH_3_-vapor-exposed sample, and the sample recovered after the NH_3_ adsorption–desorption measurement.

**8.1. Evaluation of stepwise desolvation during the Form I → Form II → Form III transformation**

TG analysis was used to evaluate the stepwise removal of DMSO during the Form I **→** Form II **→** Form III transformation. According to the SCXRD analysis, Form I contains four DMSO molecules per complete HQDPS/*t*BuA_2_ formula unit: two DMSO molecules hydrogen-bonded to the *t*BuAH^+^ cations and another two DMSO molecules hydrogen-bonded to the phenolic OH groups. Form II contains two remaining DMSO molecules associated with the phenolic OH groups, whereas Form III contains no crystallographically assigned DMSO molecules.

Based on the formula weight of Form I containing four DMSO molecules, one DMSO molecule corresponds to 8.9 wt%, two DMSO molecules correspond to 17.7 wt%, and four DMSO molecules correspond to 35.5 wt%. The TG curve of Form I showed stepwise weight-loss components of approximately 12 wt% and 17 wt% in the temperature regions associated with the first and second structural transformations, respectively (**Figure S10**). The 17 wt% component is close to the calculated weight fraction for two DMSO molecules, whereas the 12 wt% component is smaller than this ideal value. This smaller value is likely attributable to partial loss of labile DMSO molecules during sample preparation and handling, such as solvent removal, washing, and drying before the TG measurement.

Because the TG curve shows a gradual baseline drift between the two major weight-loss regions, a strict deconvolution and one-to-one assignment of each weight-loss component to a specific crystallographic DMSO site was not attempted. Nevertheless, the observation of stepwise weight loss in Form I, the disappearance of the lower-temperature component in Form II, and the absence of the corresponding DMSO-derived weight-loss components in Form III are consistent with the sequential desolvation process identified by SCXRD and PXRD. Thus, the TG profiles support the stepwise removal of crystallographically distinct DMSO molecules during the Form I **→** Form II **→** Form III transformation.

**8.2. TG analysis after DMSO-vapor exposure**

TG analysis was also carried out for the sample obtained after DMSO-vapor exposure of Form III. The reappearance of a weight-loss feature around 150 °C supported re-formation of the DMSO-containing dormant state, in agreement with the PXRD results (**Figure 4b**).

**8.3. TG analysis after NH_3_ exposure and after NH_3_ adsorption–desorption measurement**

TG measurements were performed for both the NH_3_-vapor-exposed sample and the sample recovered after the NH_3_ adsorption–desorption measurement (**Figure S20)**. These measurements were used to evaluate uptake of NH_3_-containing guest molecules and their release upon heating. For the sample exposed to NH_3_ vapor, the observed mass change supported uptake of NH_3_-containing guests while maintaining the crystal structure. For the sample recovered after the gas adsorption measurement, the weight-loss feature below 150 °C was attributed to desorption of adsorbed NH_3_, supporting relatively mild regeneration of the material.

As a control experiment, TG analysis was also performed for Form III after water-vapor exposure under otherwise similar conditions. No appreciable additional weight-loss feature corresponding to guest uptake was observed (**Figure S20**), indicating that the mass change observed after exposure to vapor from aqueous NH_3_ solution was not caused by water vapor alone.

**9. Gas Sorption Measurements**

Gas sorption measurements were performed using BELSORP-max X and BELSORP MAX instruments (MicrotracBEL Corp., Osaka, Japan). Prior to each measurement, the samples were pretreated under vacuum using the pretreatment system attached to the instrument. Unless otherwise noted, samples were degassed at 80 °C for 3 h before measurement. For NH_3_ adsorption–desorption cycling measurements, the sample was degassed at 160 °C for 90 min from the second cycle onward to ensure complete desorption of NH_3_. The sample amount was typically ca. 15–30 mg; for the remeasured low-uptake gas adsorption isotherms of Form III at 298 K, sample amounts greater than 50 mg were used to reduce the relative error. Unless otherwise noted, the pressure-equilibrium criterion was set to 300 s with a pressure variation of 0.3–0.5%.

For measurements at 298 K, adsorption isotherms were recorded up to approximately 1 bar. Gas adsorption isotherms of NH_3_, CO_2_, O_2_, H_2_, and N_2_ were measured for Forms II and III under the conditions described in the main text. In addition, low-temperature gas sorption measurements were examined for Forms II and III to evaluate pore accessibility and pore-size distribution. Because Form II showed negligible uptake under these conditions, gas-sorption-derived pore-size distribution analysis was applied only to Form III. O_2_ adsorption was measured at 195 K, whereas O_2_, H_2_, and N_2_ adsorption were measured at 77 K. The CO_2_ adsorption branch at 195 K was used for the Horvath–Kawazoe pore-size distribution analysis described in Section 9.5. Temperature-dependent NH₃ adsorption isotherms of Form III were also measured at 288, 298, and 308 K to estimate the apparent isosteric heat of adsorption, *Q*_st_, as described below and shown in **Figure S19**.

**9.1. Measurements of Forms II and III**

Gas adsorption isotherms were recorded for Forms II and III to evaluate the emergence of gas adsorption properties upon the protection–deprotection process (**Figure 7a,b**, **Table S4**). Form II was examined as the dormant state, whereas Form III was examined as the active state. Under the present conditions, NH_3_, CO_2_, O_2_, H_2_, and N_2_ were measured at 298 K. For comparison, low-temperature gas sorption isotherms of Form III are shown in **Figure S18**. CO_2_ adsorption was measured at 195 K, whereas O_2_, H_2_, and N_2_ adsorption were measured at 77 K. Under these low-temperature conditions, CO_2_ showed higher uptake than O_2_, H_2_, and N_2_.

**9.2. NH_3_ adsorption–desorption cycling measurement**

The reusability of Form III was evaluated by NH_3_ adsorption-desorption cycling measurements. Before the first cycle, the sample was pretreated at 80 °C for 3 h under vacuum. From the second cycle onward, the sample was degassed at 160 °C for 90 min before reuse in order to ensure complete desorption of NH_3_. After each cycle, the sample was regenerated and reused for the next adsorption measurement. The recovered samples were further characterized by PXRD and TG measurements. The maximum NH_3_ uptake values for each cycle are summarized in **Table S5**, and the cycling isotherms are shown in **Figure 7d**.

The NH_3_ adsorption-desorption isotherms of Form III showed a pronounced hysteresis loop. This behavior is attributed mainly to the slow release of NH_3_ retained in the phenolic-OH-lined confined pore environment. The steep uptake in the low-pressure region and the incomplete release during the desorption branch are consistent with strong but reversible host-guest interactions between NH_3_ and the pore surface. In particular, the exposed phenolic OH groups can interact with NH_3_ through O-H···N hydrogen bonding, while additional weak interactions within the confined pore environment may further stabilize the adsorbed NH_3_ molecules, as supported by the IR and DFT analyses. The nearly unchanged uptake over five cycles and the PXRD pattern after the NH_3_ adsorption-desorption measurement indicate that the hysteresis does not arise from irreversible framework degradation, but rather from delayed desorption from strongly interacting adsorption sites.

**9.3. Temperature-dependent NH₃ adsorption and *Q*_st_ analysis**

Temperature-dependent NH_3_ adsorption isotherms of Form III were measured at 288, 298, and 308 K to estimate the apparent isosteric heat of adsorption, *Q*_st_ (**Figure S19**). The *Q*_st_ values were calculated from the adsorption branches by applying the Clausius–Clapeyron relationship to pressure values interpolated at fixed NH_3_ uptakes. In this analysis, the *R*² value represents the goodness of the linear fit of ln *P* versus 1/*T* at each fixed uptake.

Because the low-pressure region of the NH_3_ isotherms showed a steep uptake, the apparent *Q*_st_ values at very low uptake are sensitive to interpolation and were not used for quantitative discussion. For Form III, one OH-equivalent uptake, defined by assuming one NH_3_ molecule per exposed phenolic OH group, corresponds to ca. 79 mL(STP) g^-1^. In the continuous region where the fitting quality was sufficiently high, corresponding approximately to 0.4–0.7 OH-equivalent, the apparent *Q*_st_ values gradually decreased from ca. 60 to ca. 40–45 kJ mol^-1^ with increasing NH_3_ uptake. These values are comparable to the *Q*_st_ values reported for NH_3_ adsorption in hydroxyl-group-containing MOFs, where such adsorption heats have been associated with appreciable host–guest interactions involving framework OH groups.^[36]^

This coverage-dependent decrease does not necessarily indicate complete saturation or disappearance of the phenolic OH sites. Rather, it suggests that the availability of readily accessible, high-affinity OH-associated local environments gradually decreases as NH_3_ uptake increases. Because Form III possesses confined pores, additional NH_3_ molecules are likely accommodated under more sterically restricted or less optimal interaction geometries, even though exposed phenolic OH groups remain present in the framework. This interpretation is consistent with energetically heterogeneous OH-containing pore environments and with the involvement of exposed phenolic OH groups as favorable NH_3_ interaction sites.

**9.4. Structural characterization after gas sorption measurements**

To evaluate structural stability during gas sorption, PXRD patterns were recorded for samples recovered after the NH_3_ adsorption–desorption measurement (**Figure 7e**). Detailed comparison of the PXRD patterns after NH_3_ uptake is described in Section 7.4. TG analysis was also carried out for the recovered sample to examine desorption behavior of the adsorbed NH_3_ (**Figure S20)**.

**9.5. Low-temperature gas sorption and pore-size distribution analysis**

The low-temperature gas sorption measurements were performed to evaluate whether gas-sorption-derived pore-size distributions could be obtained for Forms II and III. For Form II, both CO_2_ adsorption at 195 K and N_2_ adsorption at 77 K were less than 1 mL(STP) g^-1^ under the present measurement conditions. Because these uptake values are too small to provide a reliable gas-sorption-derived pore-size distribution, we did not assign a pore-size distribution to Form II. Thus, Form II was treated as the dormant, essentially nonporous state. In contrast, Form III showed measurable CO_2_ uptake at 195 K over the low-to-middle relative-pressure range. Therefore, the CO_2_-derived pore-size distribution analysis was applied only to Form III, using the CO_2_ adsorption branch at 195 K. N_2_ and O_2_ adsorption remained small in the low-to-middle relative-pressure region and increased steeply only near saturation pressure. This steep uptake near *P*_e_/*P*_0_ ≈ 1 is likely attributable to non-micropore adsorption processes, such as multilayer adsorption on the external crystal surfaces and/or condensation in interparticle spaces, rather than filling of the intrinsic crystallographic micropores. Therefore, the high-pressure uptake of N_2_ and O_2_ was not used as the basis for evaluating the intrinsic pore aperture of Form III.

The higher CO_2_ uptake compared with O_2_, H_2_, and N_2_ is not attributed primarily to the exposed phenolic OH groups, but rather to the intrinsically stronger affinity of CO_2_ toward aromatic pore environments in POS frameworks. CO_2_ has a quadrupole moment and relatively high polarizability, allowing it to interact more effectively with aromatic pore walls through quadrupolar and dispersion interactions. Similar preferential CO_2_ adsorption over N_2_, O_2_, and/or H_2_ has been reported for related POSs from our group, including stilbene-disulfonate-based and base-component-introduced POSs, where CO_2_ adsorption was rationalized in terms of interactions with aromatic pore surfaces and the molecular properties of CO_2_.^[58, 59]^ The exposed phenolic OH groups in Form III may contribute to the local polarity of the pore surface; however, the much larger enhancement observed for NH_3_ adsorption indicates that these OH groups function more specifically as NH_3_-recognition sites through hydrogen bonding. Thus, the preferential CO_2_ uptake among the general gases is best understood as arising from restricted pore diffusion combined with the relatively stronger nonspecific quadrupolar/dispersion interactions of CO_2_ with the aromatic pore walls.

The pore-size distribution was estimated from the CO_2_ adsorption branch at 195 K using the Horvath–Kawazoe method, giving a median pore width of 1.294 nm and an average pore width of 1.136 nm. Because gas-sorption-derived pore-size distributions are sensitive to the probe gas, measurement temperature, uptake amount, and analysis model, these values were used only as supplementary experimental information on pore accessibility. The geometrical pore aperture and void fraction of Form III were primarily discussed on the basis of the final crystallographic analysis and PLATON calculation.

**10. IR Spectroscopic Analysis after NH_3_ Uptake**

ATR-IR spectra were recorded to examine the interaction between the deprotected phenolic OH groups and NH_3_ in Form III. The spectra were measured for Form III before NH_3_ uptake, for the sample recovered after the NH_3_ adsorption-desorption measurement, and for the sample after NH_3_ vapor exposure (**Figure S21**).

Before NH_3_ uptake, Form III showed a broad absorption in the 3500-3000 cm^-1^ region, which is mainly assigned to phenolic O-H stretching vibrations. After NH_3_ vapor exposure, this broad absorption became more pronounced and broadened. Because this region can also include contributions from N-H stretching vibrations, the observed broadening is consistent with the formation of hydrogen-bonding interactions between the exposed phenolic OH groups and adsorbed NH_3_. In addition, the absorption around 1460 cm^-1^ increased after NH_3_ vapor exposure. This band is attributable to N-H bending vibrations of adsorbed NH_3_ or NH_3_-derived species, further supporting the presence of NH_3_ in the framework after vapor exposure.

A similar but weaker spectral change was observed for the sample recovered after the NH_3_ adsorption-desorption measurement. In this sample, the broadening in the O-H/N-H stretching region and the increase in the absorption around 1460 cm^-1^ were less pronounced than those observed after NH_3_ vapor exposure. This difference is reasonable because the sample recovered after the adsorption-desorption measurement reflects only residual NH_3_-derived species remaining after the desorption process, whereas direct NH_3_ vapor exposure affords a more extensively NH_3_-loaded state. The residual spectral changes after the adsorption-desorption measurement are consistent with the hysteretic NH_3_ adsorption behavior and incomplete desorption observed in the NH_3_ isotherm.

These IR spectral changes provide additional support that the deprotected phenolic OH groups in Form III interact with NH_3_ and function as interaction sites for NH_3_ adsorption, in agreement with the gas adsorption behavior and the DFT analysis.

**11. Diffuse Reflectance and Photoluminescence Measurements**

Steady-state photoluminescence and excitation spectra in both the solid and solution states, as well as solid-state diffuse reflectance (absorption) and external quantum yield measurements, were carried out on a JASCO FP-8500 spectrofluorometer. Solid-state diffuse reflectance data are shown as Kubelka–Munk-transformed spectra, *F*(*R*). Crystal photographs were recorded under identical lighting conditions for comparison of color changes among the different phases. Solution UV–vis absorption spectra were measured on a JASCO V-770 spectrophotometer.

Solid-state excitation and emission spectra were measured for Forms I–III (**Figures S13 and S14**). Emission spectra were recorded with an excitation wavelength of 390 nm (**Figure S14, Table S3**). Time-resolved fluorescence measurements were performed to determine the average fluorescence lifetimes (*τ*_avg_) using a HORIBA Jobin Yvon FluoroCube system equipped with a TBX Picosecond Photon Detection Module and a NanoLED-350 excitation source (peak wavelength: 352 nm) (**Figures S15–S17, Table S3**). Fluorescence lifetimes were determined by monitoring the emission at 430 nm. External quantum yields (*Φ*_ext_) were also measured for each phase (**Table S3**). These measurements were used to evaluate changes in the optical properties accompanying the Form I → Form II → Form III transformation.

Under the present measurement conditions, Forms I and II showed emission maxima at approximately 433 nm, whereas Form III showed a blue-shifted emission maximum at approximately 426 nm **(Figure S14, Table S3)**. The average fluorescence lifetimes were 0.595 ns for Form I, 0.286 ns for Form II, and 0.284 ns for Form III (**Figures S15–S17**, **Table S3**). The external quantum yield also decreased stepwise from 2.68% for Form I to 2.04% for Form II and 1.35% for Form III (**Table S3**). For evaluation of the visible response toward NH_3_, diffuse reflectance spectra were also recorded before and after exposure of Form III to vapor from 35% aqueous ammonia (**Figure 7f**).

**12. Supplementary Discussion on Morphological and Optical Changes Accompanying the SCSC Transformation**

**12.1. Morphological changes accompanying the Form I → Form II → Form III transformation**

The Form I → Form II → Form III single-crystal-to-single-crystal (SCSC) sequence was accompanied by clear macroscopic changes in crystal shape (**Figure 5**). Polarized optical microscopy showed that Form I appeared as a flat and homogeneous plate-like single crystal, whereas Form II exhibited shortening along the long-axis direction, followed by further contraction along the long axis and elongation along the short axis in Form III (**Figure 5**). These observations indicate that the anisotropic lattice deformation associated with each step of the SCSC transformation is reflected in the macroscopic crystal morphology.

In particular, the reduction in the long-axis dimension observed in the first SCSC step from Form I to Form II is consistent with the contraction of the corresponding crystallographic axis and can be understood in terms of the reorganization of the charge-assisted hydrogen-bonding (CAHB) network from a one-dimensional ladder to a two-dimensional sheet (**Figure 2**, **Table S1**). In the second SCSC step from Form II to Form III, removal of the DMSO molecules protecting the OH groups induced a more extensive framework rearrangement, including rotation and displacement of the sulfonate groups, which was accompanied by further shortening along the long axis and expansion along the short axis (**Figure 2**, **Table S1**).

Importantly, despite these pronounced changes in external crystal shape, no crystal fracture or marked collapse was observed, and single crystallinity was retained throughout the transformation (**Figure 5**). Thus, the present system enables direct correlation of microscopic structural reorganization, changes in unit-cell parameters, and macroscopic crystal-shape changes within a continuous SCSC process (**Figure 5**, **Table S1**). In this sense, the crystal-shape change is not a simple thermal deformation or crystal deterioration, but a visible manifestation of hydrogen-bond-network reorganization propagated throughout the crystal.

**12.2. Optical changes accompanying the SCSC transformation**

The three crystalline phases were also clearly distinguished by their optical responses (**Figure 6**, **Figures S13–S17**). Form I was deep red, Form II was light brown, and Form III became much paler, approaching white (**Figure 6a**). Correspondingly, the solid-state diffuse reflectance spectra showed a stepwise decrease in absorption in the visible region from Form I to Form II and then to Form III, with particularly marked suppression of the long-wavelength absorption tail (**Figure 6b**). These results indicate that the SCSC transformation is accompanied not only by structural reorganization but also by systematic modulation of the bulk optical properties.

The solid-state emission spectra also changed systematically during the phase transformation (**Figure S14)**. Forms I and II exhibited essentially the same emission maximum at approximately 433 nm, whereas Form III showed a blue-shifted emission maximum at approximately 426 nm (**Figure S14, Table S3**). The excitation spectra of Forms I and II were similar to each other, whereas Form III showed a different relative intensity distribution, indicating a change in the electronic structure associated with the second transformation step (**Figure S13)**.

Time-resolved fluorescence measurements further supported this trend (**Figures S15–S17**). The average fluorescence lifetime decreased markedly from 0.595 ns for Form I to 0.286 ns for Form II and remained nearly unchanged for Form III (0.284 ns) (**Figures S15–S17**, **Table S3**). The external quantum yield also decreased stepwise from 2.68% for Form I to 2.04% for Form II and 1.35% for Form III (**Table S3**). Notably, the substantial decrease in lifetime and quantum yield from Form I to Form II occurred without a significant shift in the emission maximum, suggesting that enhancement of nonradiative deactivation pathways, rather than a major change in emission energy, is responsible for the reduced emission efficiency in this step.

By contrast, the blue shift observed from Form II to Form III is reasonably attributed to the change in the hydrogen-bonding environment around HQDPS upon removal of the DMSO molecules protecting the OH groups. Accordingly, the optical changes are best understood as consequences of the stepwise reorganization of the CAHB network and the local hydrogen-bonding environment around the OH groups, rather than being dominated by π–π stacking effects. Importantly, these optical responses were observed for well-defined crystalline phases retaining single crystallinity, demonstrating that the hydrogen-bonding changes can be directly correlated with changes in electronic state and optical properties in the present SCSC system.

**13. Supplementary Figures and Tables**

**Figure S6.** Crystal structures of Form I, Form II, and Form III.

**Figure S7.** Local hydrogen-bonding environments of crystallographically nonequivalent DMSO molecules in Form I.

**Figure S8**. Comparison of the crystal structure obtained from MeOH recrystallization and that of Form I obtained from DMSO recrystallization.

**Figure S9**. Crystal structures and charge-assisted hydrogen-bonding network motifs of HQDPS/*t*BuA Form I and TPDS-OMe₂/*t*BuA Forms I and II.

**Figure S10**. TG curve of Form I showing stepwise weight losses associated with the desolvation of crystallographically distinct DMSO molecules.

**Figure S11**. Pore-shape analysis of Form III based on the crystallographic disorder of the central benzene ring of the terphenyl moiety. (a) One orientation provides an apparent open channel with an aperture of ca. 6.7 × 4.0 Å. (b) The other orientation protrudes into the channel and narrows the local bottleneck aperture to ca. 1.8 Å.

**Figure S12**. Structural comparison of DMA-recrystallized HQDPS/*t*BuA and HQDPS/*t*BuA Form II.

**Figure S13.** Solid-state excitation spectra of Forms I–III.

**Figure S14.** Solid-state emission spectra of Forms I–III.

**Figure S15**. Time-resolved fluorescence decay profiles of Form I.

**Figure S16**. Time-resolved fluorescence decay profiles of Form II.

**Figure S17.** Time-resolved fluorescence decay profiles of Form III.

**Figure S18**. Low-temperature gas sorption analysis of Form III. (a) Gas sorption isotherms of Form III for CO_2_ at 195 K and for O_2_, H_2_, and N_2_ at 77 K, plotted as adsorbed volume versus relative pressure (*P*_e_/*P*_0_). Filled and open symbols represent the adsorption and desorption branches, respectively. (b) CO_2_-derived HK pore-size distribution of Form III estimated from the CO_2_ adsorption branch at 195 K.

**Figure S19**. Temperature-dependent NH_3_ adsorption isotherms and isosteric heat of adsorption for Form III. (a) NH_3_ adsorption isotherms measured at 288, 298, and 308 K. (b) Apparent isosteric heat of NH₃ adsorption, *Q*_st_, estimated from the temperature-dependent adsorption isotherms as a function of NH_3_ uptake. The color classification indicates the fitting reliability based on the *R*² values obtained from the linear fit of ln *P* versus 1/*T* at each fixed uptake.

**Figure S20**. TG curves of Form III after NH_3_ adsorption-desorption measurement, after NH_3_ vapor exposure, after reactivation, and after H_2_O vapor exposure. (a) NH_3_-containing samples and the reactivated sample. (b) Sample after H_2_O vapor exposure under otherwise similar conditions.

**Figure S21**. ATR-IR spectra of Form III before NH_3_ uptake, after the NH_3_ adsorption-desorption measurement, and after NH_3_ vapor exposure. The highlighted regions correspond to O-H/N-H stretching vibrations and N-H bending vibrations.

**Figure S22**. Comparison of possible Form III framework models composed of four framework molecules and their calculated relative energies. The relative energy (ΔE) was calculated with respect to the most stable AB-AB model.

**Figure S23**. Initial and optimized structures for NH_3_ adsorption models in Form III. NH_3_ molecules were initially placed near the *t*BuAH^+^ cation, phenolic OH group, or SO_3_^⁻^ moiety of the Form III framework model. Geometry optimization showed that NH_3_ is stabilized within the confined pocket, with the phenolic OH region providing a favorable adsorption environment.

**Figure S24**. Theoretical analysis of the NH_3_ adsorption site in Form III. (a) Electrostatic potential map of NH_3_, showing the electron-rich region around the N atom. (b) Inclusion space and electrostatic potential maps of the Form III framework model composed of four framework molecules. The electron-deficient region in the confined pocket is highlighted as a favorable interaction site for NH_3_. (c) Optimized NH_3_ adsorption structure in the Form III framework model, showing stabilization of NH_3_ within the confined pocket near the phenolic OH groups. (d) Noncovalent interaction (NCI) plot of the optimized NH_3_ adsorption structure. The NCI analysis supports cooperative stabilization of NH_3_ through O-H···N hydrogen bonding and additional C-H···N and van der Waals interactions within the electrostatically favorable confined pocket.

**Table S1**. Crystallographic data and refinement details for Forms I–III.

| **Crystal** | **Form I** | **Form II** | **Form III** |
| --- | --- | --- | --- |
| **Crystal system** | **triclinic** | **triclinic** | **monoclinic** |
| **Space group** | ***P*-1 (#2)** | ***P*-1 (#2)** | ***P*_2_/c (#13)** |
| ***a* (Å)** | **6.2207(2)** | **6.2412(2)** | **16.318(3)** |
| ***b* (Å)** | **8.6287(3)** | **8.5653(3)** | **10.0660(12)** |
| ***c* (Å)** | **21.6463(5)** | **17.7117(7)** | **10.9587(15)** |
| ***α* (°)** | **89.785(2)** | **85.531(3)** | **90** |
| ***β* (°)** | **88.728(2)** | **80.410(3)** | **108.973(16)** |
| ***γ* (°)** | **78.295(2)** | **79.263(3)** | **90** |
| ***V* (Å^3^)** | **1137.46(6)** | **916.18(6)** | **1702.3(4)** |
| ***R*1** | **0.0681** | **0.0442** | **0.1714** |
| ***wR*2** | **0.2401** | **0.1438** | **0.4648** |
| **GOF** | **1.690** | **0.989** | **1.443** |
| **Completeness** | **0.974** | **0.990** | **0.996** |
| **Temperature (K)** | **298** | **298** | **293** |
| **CCDC number** | **2552373** | **2552375** | **2552376** |

**Table S2**. Crystallographic data and refinement details for the control and additional recrystallization structures: the control crystal obtained by recrystallization of the HQDPS/*t*BuA salt from methanol, TPDS-OMe_2_/*t*BuA Forms I and II, and DMA-recrystallized HQDPS/*t*BuA.

| **Crystal** | **HQDPS/*t*BuA**  **from MeOH** | **TPDS-OMe_2_/*t*BuA Form I** | **TPDS-OMe_2_/*t*BuA**  **Form II** | **DMA-recrystallized HQDPS/*t*BuA** |
| --- | --- | --- | --- | --- |
| **Crystal system** | **monoclinic** | **triclinic** | **triclinic** | **monoclinic** |
| **Space group** | ***P*2_1_/*c* (#14)** | ***P*-1 (#2)** | ***P*-1 (#2)** | ***P*21/*n* (#14)** |
| ***a* (Å)** | **7.1062(2)** | **6.2318(2)** | **11.8731(5)** | **17.5016(12)** |
| ***b* (Å)** | **12.5396(3)** | **18.4171(6)** | **13.0891(4)** | **6.5666(3)** |
| ***c* (Å)** | **17.6152(4)** | **19.3659(7)** | **20.7198(6)** | **18.9299(12)** |
| ***α* (°)** | **90** | **87.348(3)** | **81.056(3)** | **90** |
| ***β* (°)** | **97.919(2)** | **87.101(3)** | **84.158(3)** | **110.908(8)** |
| ***γ* (°)** | **90** | **88.026(3)** | **85.002(3)** | **90** |
| ***V* (Å^3^)** | **1554.70(7)** | **2216.29(13)** | **3156.02(19)** | **2032.3(2)** |
| ***R*1** | **0.0544** | **0.1107** | **0.0838** | **0.0741** |
| ***wR*2** | **0.1768** | **0.3267** | **0.3277** | **0.2096** |
| **GOF** | **1.233** | **1.065** | **1.095** | **1.165** |
| **Completeness** | **0.999** | **0.992** | **0.996** | **0.999** |
| **Temperature (K)** | **293** | **213** | **213** | **213** |
| **CCDC number** | **2552377** | **2562125** | **2562126** | **2562127** |

**Table S3.** Optical properties of Forms I–III, including emission maxima (*λ*_em,max_), average fluorescence lifetimes (*τ*_avg_), and external quantum yields (*Φ*_ext_).

|  | **Form I** | **Form II** | **Form III** |
| --- | --- | --- | --- |
| ***λ*_em,max_ / nm** | **433** | **433** | **426** |
| ***τ*_avg_ / ns** | **0.595** | **0.286** | **0.284** |
| ***Φ*_ext_ / %** | **2.68** | **2.04** | **1.35** |

**Table S4.** Gas adsorption data of Forms II and III at 298 K near 1 bar. Values were taken from the adsorption branches at the highest pressure points measured for each gas. The NH_3_/CO_2_ uptake ratio is also shown.

|  | **NH_3_ uptake / mL(STP) g^-1^** | **CO_2_ uptake / mL(STP) g^-1^** | **O_2_ uptake / mL(STP) g^-1^** | **H_2_ uptake / mL(STP) g^-1^** | **N_2_ uptake / mL(STP) g^-1^** | **NH_3_/CO_2_ uptake ratio** |
| --- | --- | --- | --- | --- | --- | --- |
| **Form II** | **10.2** | **2.5** | **0.6** | **0.1** | **0.3** | **4.0** |
| **Form III** | **89.2** | **3.0** | **0.5** | **0.0** | **0.1** | **29.8** |

**Table S5.** Maximum NH_3_ uptake of Form III in five adsorption–desorption cycles at 298 K. The values were taken from the adsorption branches at the highest pressure points measured in each cycle.

|  | **Cycle 1** | **Cycle 2** | **Cycle 3** | **Cycle 4** | **Cycle 5** |
| --- | --- | --- | --- | --- | --- |
| **NH_3_ uptake / mL(STP) g^-1^** | **87.1** | **89.2** | **88.2** | **87.8** | **85.5** |

**Table S6**. Reference electronic energies of the AB-AB Form III framework model, isolated NH_3_, and the corresponding combined system used for NH_3_ adsorption-site analysis.

| **Molecule** | **Energy / Hartree** | **Energy / kcal mol^-1^** |
| --- | --- | --- |
| **Framework III AB-AB** | **−10070.638169** | **−6,319,420.86** |
| **NH_3_** | **−56.4731810158** | **−35,437.429** |
| **Framework III AB-AB + NH_3_** | **−10127.1113500158** | **−6,354,858.289** |

**Table S7**. Calculated electronic energies and adsorption energies of optimized NH_3_ adsorption models obtained from different initial NH_3_ positions in the Form III framework. NH_3_ was initially placed near the *t*BuAH^+^ cation, phenolic OH group, or SO_3_^−^ moiety of the AB-AB Form III framework model. The adsorption energy was calculated using the isolated AB-AB Form III framework model and isolated NH_3_ as reference states. The relative energy (ΔE) was calculated with respect to the lowest-energy optimized model.

| **Initial NH_3_ position** | **Energy**  **/ Hartree** | **Energy**  **/ kcal mol^-1^** | **Adsorption energy**  **/ kcal mol^-1^** | **ΔE**  **/ kcal mol^-1^** |
| --- | --- | --- | --- | --- |
| ***t*BuAH^+^ (A)** | **−10127.136906** | **−6,354,889.36** | **−31.1** | **0** |
| **Phenolic OH (A)** | **−10127.136906** | **−6,354,889.36** | **−31.1** | **0** |
| **SO_3_^-^ (B)** | **−10127.136754** | **−6,354,889.26** | **−31.0** | **+0.1** |

**14. Crystallographic Data**

Deposition numbers 2552373 (for Form I), 2552375 (for Form II), 2552376 (for Form III), 2552377 (for the control crystal obtained by recrystallization of the HQDPS/*t*BuA salt from methanol), 2562125 (for TPDS-OMe_2_/*t*BuA Form I), 2562126 (for TPDS-OMe_2_/*t*BuA Form II), and 2562127 (for DMA-recrystallized HQDPS/*t*BuA) contain the supplementary crystallographic data for this paper. These data are provided free of charge by the Cambridge Crystallographic Data Centre.

Form III corresponds to the activated phase obtained after removal of the DMSO molecules that had supramolecularly protected the phenolic OH groups. Because this phase contains significant disorder and solvent-accessible voids, diffuse residual electron density remained in the pore region and was treated using the SQUEEZE procedure implemented in PLATON. Although the diffraction quality is limited, the model converged satisfactorily after SQUEEZE treatment and provides a reasonable representation of the overall framework of the activated phase.

**15. Computational Methods**

Quantum chemical calculations for the Form III NH_3_ adsorption study were performed using Gaussian 16, Revision C.02,^[64]^ on the Numerical Materials Simulator at NIMS. Geometry optimizations were carried out at the M06-2X/def2-SVP level of theory based on the Form III crystal structure. To address the disorder of the phenolic OH groups in the Form III crystal structure, four possible OH arrangements were examined using cluster models based on the experimentally determined Form III structure. Geometry optimizations were carried out while fixing the framework atoms at their crystallographic positions and allowing the selected hydrogen atoms to relax. The most stable OH arrangement was then selected as the representative framework structure for the subsequent NH_3_ adsorption-site calculations. Then, to evaluate the possible adsorption sites of NH_3_ in the crystalline framework, structural models were constructed by placing NH_3_ near the possible interaction sites: (i) phenolic OH groups, (ii) SO_3_⁻ moieties, and (iii) the *t*BuAH^+^ cation. During these geometry optimizations, the framework atoms were fixed at the previously obtained representative structure, whereas the NH_3_ molecule was allowed to relax. The SCF convergence was assisted using the XQC algorithm when necessary. No symmetry constraints were imposed in the calculations. The relative stability of each adsorption model was evaluated from the optimized electronic energies. Electrostatic potential analyses and noncovalent interaction analyses, including NCI plots, were performed using the optimized structures to visualize the local electrostatic environment and weak host–guest interactions around the adsorbed NH_3_ molecule.

**16. References**

[58]	A. Yamamoto, T. Hirukawa, I. Hisaki, M. Miyata, N. Tohnai, “Multifunctionalized porosity in zeolitic diamondoid porous organic salt: selective adsorption and guest-responsive fluorescent properties” *Tetrahedron Letters* **54**, (2013): 1268-1273.

[59]	T. Ami, K. Oka, K. Tsuchiya, W. Kosaka, H. Miyasaka, N. Tohnai, “The introduction of a base component to porous organic salts and their CO_2_ storage capability” *CrystEngComm* **25**, (2023): 2321-2325.

[60]	O. V. Dolomanov, L. J. Bourhis, R. J. Gildea, J. A. K. Howard, H. Puschmann, “OLEX2: a complete structure solution, refinement and analysis program” *Journal of Applied Crystallography* **42**, (2009): 339-341.

[61]	G. M. Sheldrick, “SHELXT - integrated space-group and crystal-structure determination” *Acta Crystallogr A Found Adv* **71**, (2015): 3-8.

[62]	G. M. Sheldrick, “Crystal structure refinement with SHELXL” *Acta Crystallogr C Struct Chem* **71**, (2015): 3-8.

[63]	A. L. Spek, “PLATON SQUEEZE: a tool for the calculation of the disordered solvent contribution to the calculated structure factors” *Acta Crystallogr C Struct Chem* **71**, (2015): 9-18.

[64]	Gaussian 16, Revision C.02, M. J. Frisch, G. W. Trucks, H. B. Schlegel, G. E. Scuseria, M. A. Robb, J. R. Cheeseman, G. Scalmani, V. Barone, G. A. Peters, H. Nakatsuji, X. Li, M. Caricato, A. V. Marenich, J. Bloino, B. G. Janesko, R. Gomperts, B. Mennucci, H. P. Hratchian, J. V. Ortiz, A. F. Izmaylov, J. L. Sonnenberg, D. Williams-Young, F. Ding, F. Lipparini, F. Egidi, J. Goings, B. Peng, A. Petrone, T. Henderson, D. Ranasinghe, V. G. Zakrzewski, J. Gao, N. Rega, G. Zheng, W. Liang, M. Hada, M. Ehara, K. Toyota, R. Fukuda, J. Hasegawa, M. Ishida, T. Nakajima, Y. Honda, O. Kitao, H. Nakai, T. Vreven, K. Throssell, J. A. Montgomery, Jr., J. E. Peralta, F. Ogliaro, M. J. Bearpark, J. J. Heyd, E. N. Brothers, K. N. Kudin, V. N. Staroverov, T. A. Keith, R. Kobayashi, J. Normand, K. Raghavachari, A. P. Rendell, J. C. Burant, S. S. Iyengar, J. Tomasi, M. Cossi, J. M. Millam, M. Klene, C. Adamo, R. Cammi, J. W. Ochterski, R. L. Martin, K. Morokuma, O. Farkas, J. B. Foresman, and D. J. Fox, Gaussian, Inc., Wallingford CT, 2016.
